# Comparative efficacy of digital health interventions and traditional mind-body exercises for pulmonary rehabilitation in chronic obstructive pulmonary disease: a systematic review and network meta-analysis

**DOI:** 10.3389/fmed.2026.1871103

**Published:** 2026-08-24

**Authors:** Kunning Wei, Xiaoyan Liu, Bowen Luo, Haiyan Wang, Haoyu Su, Chunli Mei

**Affiliations:** 1 School of Nursing, Beihua University, Jilin City, China; 2 The Third Clinical College, Shanxi University of Traditional Chinese Medicine, Taiyuan, China

**Keywords:** chronic obstructive pulmonary disease, digital health interventions, network meta-analysis, pulmonary rehabilitation, traditional mind-body exercises

## Abstract

**Background:**

Digital health interventions (DHIs) and traditional mind-body exercises (TMBEs) have shown potential benefits for pulmonary rehabilitation in stable COPD, but their comparative efficacy remains unclear.

**Objective:**

To compare DHIs (Synchronous telerehabilitation, Composite digital interventions) and TMBEs (Tai Chi, Baduanjin, Yoga) for stable COPD using network meta-analysis.

**Methods:**

PubMed, Embase, Cochrane Library, Web of Science, CNKI, Wanfang, and VIP were searched from inception to April 2026. RCTs comparing DHIs or TMBEs with usual care were included. Bayesian network meta-analysis and network meta-regression were performed. Outcomes included 6MWD, FEV1, FVC, SGRQ and CAT. Treatment effects were expressed as mean differences (MDs) with 95% credible intervals (CrIs), and treatment ranking probabilities and certainty of evidence were assessed using SUCRA and GRADE, respectively.

**Results:**

Forty-seven RCTs enrolling 4,720 stable COPD patients were included. Compared with usual care, Baduanjin and Yoga significantly elevated 6MWD, FEV1 and FVC. Tai Chi and Baduanjin produced meaningful SGRQ reductions; four interventions (Baduanjin, Composite digital interventions, Synchronous telerehabilitation, Tai Chi) yielded significant CAT score improvements. SUCRA results revealed Yoga possessed the highest likelihood of top ranking for exercise capacity (6MWD:82.9%) and lung function (FEV1: 90.5%; FVC: 100.0%), with Baduanjin second for FVC (74.8%). Tai Chi ranked best for quality-of-life indicators (SGRQ: 80.7%; CAT: 92.3%). These rankings stemmed mostly from indirect comparisons and cannot confirm definitive superiority between active interventions. Meta-regression indicated longer intervention duration correlated with larger 6MWD improvements.

**Conclusion:**

Targeted mind-body exercises and synchronous telerehabilitation exert beneficial effects vs. usual care for stable COPD. Yoga is most likely optimal for exercise endurance and lung function, while Tai Chi performs best for symptom-related quality of life. These rankings rely heavily on indirect evidence and fail to prove clear superiority of any single intervention. Baduanjin shows consistent benefits across all measured outcomes; Synchronous telerehabilitation serves as a viable choice for alleviating respiratory symptoms. Composite digital interventions require more high-quality trials to verify their clinical value.

**Systematic review registration:**

https://www.crd.york.ac.uk/prospero/display_record.php?RecordID=261380778, Identifier: CRD420261380778

## 1 Introduction

Chronic obstructive pulmonary disease (COPD) is a common, preventable, and treatable progressive and heterogeneous respiratory disease characterized by persistent airflow limitation (28). It is often accompanied by progressively worsening respiratory symptoms and declining lung function, severely impairing patients' ability to perform daily activities and their quality of life (29). According to the World Health Organization, chronic respiratory diseases rank as the third leading cause of death globally, with COPD being the primary lethal subtype, accounting for approximately 3.7 million deaths annually (30). The global burden of COPD is currently substantial, with approximately 480 million people affected in 2020; the latest modeling studies predict that this number will rise to 592 million by 2050, representing a relative increase of 23.3%. Without effective prevention and rehabilitation interventions, the prevalence of the disease will continue to climb, further exacerbating the pressure on global public health systems (30). The Global Initiative for Chronic Obstructive Lung Disease (GOLD) lists pulmonary rehabilitation programs as a standard component of treatment for COPD patients and emphasizes that exercise training is central to these programs, as it can reduce the frequency of acute exacerbations, lower the incidence of complications, and improve quality of life (31, 32). However, traditional center-based pulmonary rehabilitation faces challenges such as low participation rates, poor adherence, and limited geographical accessibility; globally, fewer than 2% of eligible COPD patients have access to pulmonary rehabilitation services (33).

To overcome these obstacles, two key models of pulmonary rehabilitation have emerged in recent years: one is digital health interventions, including tele-rehabilitation, mobile health applications, and wearable devices, which extend pulmonary rehabilitation services into patients' home environments (34). The other category consists of traditional mind-body exercises, such as Tai Chi, Baduanjin, and Yoga. These low-intensity forms of exercise integrate breathing control, physical postures, and elements of meditation, making them particularly suitable for COPD patients with limited exercise tolerance (35, 36). The GOLD 2025 guidelines have formally recognized the value of telemedicine and alternative exercise modalities in pulmonary rehabilitation (37).

However, there is currently a lack of comparative studies on the relative effectiveness of these five rehabilitation modalities (Synchronous telerehabilitation, Composite digital interventions, Tai Chi, Baduanjin, and Yoga). Therefore, this study employs a network meta-analysis to systematically evaluate the rehabilitation outcomes of the aforementioned digital health interventions and traditional mind-body exercises for patients with COPD, with the aim of providing evidence-based support for clinical decision-making.

## 2 Methods

This study strictly adheres to the PRISMA-NMA reporting guidelines, and the study protocol has been registered on the PROSPERO international systematic review registry (registration number: CRD420261380778). All analytical data are derived from published randomized controlled trials and do not involve raw patient data; therefore, no ethical approval is required.

### 2.1 Inclusion criteria

#### 2.1.1 Study population

Patients with stable COPD diagnosed according to GOLD criteria (38) (post-bronchodilator forced expiratory volume in one second (FEV_1_) / forced vital capacity (FVC) < 0.70), with no restrictions on GOLD stage (Stages I–IV) regardless of previous history of exacerbations.

#### 2.1.2 Intervention

The experimental group received a digital health intervention (Synchronous telerehabilitation, Composite digital interventions) or traditional mind-body exercises (Tai Chi, Baduanjin, Yoga), with a clear and comprehensive intervention protocol; In this study, Synchronous telerehabilitation was defined as digitally delivered rehabilitation programs involving real-time interaction and supervision between patients and healthcare professionals. Composite digital interventions were defined as digitally supported rehabilitation programs integrating one or more digital components, such as mobile applications, wearable devices, online self-management platforms, automated feedback systems, or asynchronous digital support, without requiring continuous real-time supervision. The control group received usual care (UC), which was defined as the standard management provided to patients with COPD without the investigated rehabilitation interventions. Depending on the individual study protocols, UC could include health education, pharmacological treatment, routine nursing care, breathing exercises, and general physical activity recommendations. Because usual care reflected routine clinical practice, its specific components varied across studies; however, all UC groups served as the common comparator representing the absence of the evaluated rehabilitation modalities.

#### 2.1.3 Outcome measures

At least one of the following outcome measures must be reported: exercise capacity measured by 6-min walk distance (6MWD); forced expiratory volume in one second (FEV_1_) and forced vital capacity (FVC); and quality of life measured by the St. George's Respiratory Questionnaire (SGRQ) and the Chronic Obstructive Pulmonary Disease Assessment Test (CAT).

#### 2.1.4 Study design

Randomized controlled trials; no restrictions on language or publication status.

### 2.2 Exclusion criteria

- Participants with a history of comorbidities (e.g., malignant tumors, symptomatic cardiovascular disease);
- Studies lacking specific data or statistical data;
- Participants who were simultaneously enrolled in other studies;
- Studies with significant inconsistencies in baseline data.

### 2.3 Literature search strategy

A systematic search was conducted in four English-language databases (PubMed, Embase, Cochrane Library, and Web of Science) and three Chinese-language databases (CNKI, Wangfang, and VIP). The search period spanned from the inception of each database to April 23, 2026. The search utilized a combination of subject headings and free-text terms. The following medical keywords were used in this search: “Chronic Obstructive Pulmonary Disease”, “Telerehabilitation”, “Digital Health”, “Tai Chi”, “Yoga” and “Baduanjin”. For details on the database search strategy, see Supplementary Table S1.

### 2.4 Study selection

Study selection was conducted independently by two reviewers. After deduplicating all retrieved records using EndNote 21, they were screened in sequence based on titles / abstracts and full texts. The two reviewers independently completed each stage of the process in accordance with the inclusion and exclusion criteria. When full-text documents were unavailable or data were missing, the original authors were contacted to request them. Disagreements between reviewers were resolved through discussion or third-party arbitration.

### 2.5 Data extraction

Two review authors independently extracted data using a standardized form. Any disagreements were resolved through consensus or discussion with a third review author. The extracted information included the following: first author's name (country), sample size, mean age, GOLD classification, rehabilitation interventions, duration of intervention, and outcome measures (mean and standard deviation). For pulmonary function outcomes (FEV_1_ and FVC), measurement units were checked and standardized across all included studies. When values were reported in milliliters (ml), they were converted to liters (L) before statistical analysis (1 L = 1,000 ml) to ensure consistency across studies.

The following outcomes were analyzed: (1) lung function, such as FEV_1_ and FVC; (2) exercise capacity, such as 6MWD; (3) health status, as measured by the CAT score; and (4) quality of life, as measured by the SGRQ.

### 2.6 Risk of bias assessment

Two reviewers assessed independently the risk of bias in the included trials using the Cochrane Risk of Bias Assessment Tool. The assessment covered the following seven domains: (1) random sequence generation; (2) allocation concealment; (3) blinding of participants and researchers; (4) blinding of outcome assessment; (5) incomplete outcome data; (6) selective reporting of results; (7) Other potential sources of bias. The risk of bias in each domain will be rated as “low risk”, “high risk”, or “unclear risk”. Any disagreements between reviewers will be resolved through discussion or, if necessary, by arbitration from a third reviewer.

### 2.7 Statistical analysis

The risk of bias assessment was performed using the Cochrane Risk of Bias Assessment Tool. RevMan 5.3 (Review Manager) was used only for visualization and presentation of the risk-of-bias results. Pairwise meta-analyses were first conducted to evaluate direct comparisons between each intervention and UC. The I^2^ statistic was used to assess heterogeneity in pairwise meta-analyses. If I^2^ ≤ 50%, a fixed-effects model was applied; if I^2^ > 50%, a random-effects model was applied. Subsequently, Bayesian network meta-analysis was performed to integrate both direct and indirect evidence across different rehabilitation interventions. R 4.4.1 and Stata 18.0 were used to perform Bayesian network meta-analysis and generate comparison-adjusted funnel plots. A Bayesian random-effects model was applied to account for potential between-study heterogeneity across the treatment network. Since all outcome measures in this study were continuous variables, the mean differences (MDs) with 95% credible intervals (CrIs) were used as effect estimates. For pulmonary function outcomes (FEV1 and FVC), all effect estimates were calculated using liters (L) as the standardized unit. If the 95% CrI did not include 0, the difference was considered statistically distinguishable; if it included 0, the difference was considered not statistically distinguishable. UC was specified as the reference treatment in the network meta-analysis. To ensure consistency in the interpretation of treatment effects, outcome directions were harmonized before analysis when necessary. For 6MWD, FEV_1_, and FVC, higher values indicate greater clinical improvement, whereas for the SGRQ and CAT, lower values indicate greater clinical improvement. Therefore, the interpretation of MD values was based on the clinical direction of each outcome rather than the numerical sign alone. Potential sources of heterogeneity were further investigated using network meta-regression analyses for outcomes with sufficient numbers of included studies. Intervention duration (weeks), publication year, and sample size were included as study-level covariates. The impact of these variables on treatment effects was evaluated using regression coefficients and 95% credible intervals (CrIs). A covariate was considered statistically distinguishable if its 95% CrI did not include zero. The transitivity assumption was assessed before performing network meta-analysis by examining the similarity of important clinical and methodological characteristics across studies, including baseline disease severity, patient characteristics, intervention duration, and outcome measurements. The variability in usual care definitions across studies was also considered during this assessment. Although UC components differed between trials, all control groups represented routine COPD management without the investigated rehabilitation interventions, supporting their inclusion as a common comparator. For digital health interventions, the potential variability in intervention components, delivery modes, supervision intensity, and patient engagement was considered during the assessment of clinical heterogeneity and transitivity. Global model fit and between-study heterogeneity were evaluated by comparing the Deviance Information Criterion (DIC) between the consistency model and the unrelated mean effects model. A difference in DIC of less than 5 was considered to indicate no important difference in model fit between the two models. The efficacy of each intervention was ranked using the Surface Under the Cumulative Ranking Curve (SUCRA). A higher SUCRA value indicates a greater probability that an intervention ranks among the most effective treatments. However, SUCRA values represent relative ranking probabilities and should not be interpreted as definitive evidence of treatment superiority. Comparison-adjusted funnel plots were used to assess potential publication bias. Effect estimates and treatment rankings were interpreted according to the clinical direction of each outcome measure. The certainty of evidence for primary outcomes was graded using the GRADE (Grading of Recommendations Assessment, Development, and Evaluation) approach, considering risk of bias, inconsistency, indirectness, imprecision, and publication bias.

## 3 Results

### 3.1 Literature search results

A total of 10,468 records were identified through database searching. After removing duplicates (*n* =4,893), records excluded by automated tools (n = 809), and other ineligible records (*n* = 368), 4,398 records remained for screening. During the title and abstract screening phase, 4,088 records were excluded due to not meeting the inclusion criteria, leaving 310 articles for full-text assessment. After full-text review, 263 studies were excluded for reasons of ineligibility, and 47 studies ultimately met the inclusion criteria and were included in this systematic review (1–25, 39–60). The detailed study selection process and reasons for exclusion are presented in Supplementary Figure S1.

### 3.2 Characteristics of the included studies

This study included a total of 47 randomized controlled trials involving 4,720 patients with stable COPD. The overall mean age was approximately 64.5 years. Of these, 40 studies explicitly reported gender distribution, including 2,583 men and 1,556 women; the remaining 7 studies did not provide detailed reports on gender distribution. Regarding outcome measures, 25 studies reported 6MWD, 31 reported FEV_1_, 23 reported FVC, 15 reported SGRQ, and 20 reported CAT (see Table 1). For pulmonary function outcomes, the included studies reporting FEV_1_ and FVC presented values either directly in liters (L) or in milliliters (ml). Values reported in milliliters were converted into liters before statistical analysis to ensure consistency across studies.

**Table 1:** Baseline characteristics of the 51 studies included in this network meta-analysis.

References	Country	Sample size (M/F, *n*)		Average age (year)		GOLD grading	Rehabilitation interventions		Intervention time	Outcome indicators
		E	C	E	C		E	C		
Jiechao (2025) (1)	China	20 (7/13)	25 (15/10)	69.20 ± 3.97	68.56 ± 4.83	GOLD I- III	Baduanjin	UC	12weeks	FEV_1_, FVC, CAT
Vorrink et al. (47)	Netherlands	84 (42/42)	73 (37/36)	62 ± 9	63 ± 8	GOLD II- III	Composite digital interventions	UC	24 weeks	6MWD, FEV_1_, FVC
Tupper et al. (55)	Denmark	141 (55/86)	140 (76/64)	69.8 ± 9.0	69.4 ± 10.1	GOLD II- IV	Synchronous telerehabilitation	UC	24 weeks	CAT
Saleh et al. (40)	Norway	57 (15/42)	57 (26/31)	69.0 ± 1.17	68.07 ± 1.13	GOLD III- IV	Synchronous telerehabilitation	UC	2 weeks	FEV_1_
Papp et al. (49)	Sweden	19 (5/14)	17 (7/10)	40–76	43–86	GOLD I- III	Yoga	UC	12 weeks	6MWT, FEV_1_, FVC
Moy et al. (42)	USA	36 (17/19)	37 (27/10)	69.6 ± 7.5	70.5 ± 9.2	GOLD I- IV	Tai Chi	UC	24 weeks	6MWT
Luo et al. (51)	China	108 (76/32)	108 (74/34)	68.03 ± 6.58	67.43 ± 7.34	GOLD II- III	Tai Chi	UC	52 weeks	FEV_1_, FVC, SGRQ
Kraemer et al. (43)	USA	61 (43/18)	31 (18/13)	68.6 ± 9.2	67.5 ± 7.7	GOLD II- IV	Tai Chi	UC	12 weeks	6MWT
Køpfli et al. (54)	Denmark	101 (35/66)	97 (42/55)	69 ± 8.3	70 ± 7.8	GOLD III- IV	Synchronous telerehabilitation	UC	24 weeks	SGRQ
Kantatong et al. (50)	Thailand	25 (15/10)	25 (19/6)	69.68 ± 7.67	67.48 ± 10.17	GOLD I- II	Tai Chi	UC	24 weeks	6MWT, FEV_1_, FVC, SGRQ
Kaminsky et al. (53)	USA	21 (7/14)	22 (10/12)	68 ± 7	68 ± 9	GOLD II- IV	Yoga	UC	12 weeks	6MWD, FEV_1_, SGRQ, CAT
Jiang et al. (46)	China	53 (44/9)	53 (43/10)	70.92 ± 6.38	71.83 ± 7.60	GOLD II- IV	Composite digital interventions	UC	12 weeks	CAT, SGRQ
Huang et al. (52)	China	51 (44/7)	51 (44/7)	73.31 ± 6.16	74.92 ± 4.96	GOLD II- IV	Composite digital interventions	UC	12 weeks	CAT
Gloeckl et al. (39)	Germany	136 (71/65)	142 (70/72)	60–72	59–70	GOLD I- III	Composite digital interventions	UC	12 weeks	CAT
Dogan and Kokturk (45)	Turkey	37 (27/10)	37 (28/9)	49–89	55–89	GOLD III- IV	Synchronous telerehabilitation	UC	12 weeks	CAT
Zhu et al. (35)	China	63 (36/27)	63 (36/27)	69.0 ± 8.7	68.0 ± 9.2	NR	Baduanjin	UC	24 weeks	6MWD, SGRQ
Zhang (57)	China	42 (32/10)	40 (33/7)	62.12 ± 6.8	63.3 ± 6.07	GOLD I- IV	Composite digital interventions	UC	12 weeks	FEV_1_, FVC, SGRQ
Zhang et al. (16)	China	48 (26/22)	48 (29/19)	52–73	51–75	NR	Baduanjin	UC	8 weeks	FEV_1_, FVC, CAT
Zhang et al. (17)	China	30 (17/13)	30 (18/12)	65.46 ± 6.74	64.82 ± 6.23	GOLD I- III	Baduanjin	UC	24 weeks	6MWT, FEV_1_, FVC
Eini (58)	China	21 (13/8)	21 (11/10)	65.0 ± 7.03	68.8 ± 7.11	GOLD I- III	Tai Chi	UC	12 weeks	6MWT, FEV_1_, FVC, CAT
Cai et al. (22)	China	24 (21/3)	28 (25/3)	70.67 ± 8.79	71.29 ± 10.08	NR	Baduanjin	UC	12 weeks	6MWT, FEV_1_, FVC, CAT
Chen et al. (23)	China	100 (85/15)	100 (82/18)	64.24 ± 8.24	65.24 ± 8.34	NR	Baduanjin	UC	12 weeks	6MWD, FEV_1_, FVC, SGRQ, CAT
Zhang et al. (56)	China	39 (27/12)	40 (26/14)	67.82 ± 6.13	68.23 ± 6.46	GOLD II- IV	Synchronous telerehabilitation	UC	12 weeks	6MWD, FEV_1_, SGRQ, CAT
Yu et al. (5)	China	73 (44/29)	72 (41/31)	74.6 ± 6.1	75.2 ± 5.9	NR	Synchronous telerehabilitation	UC	24 weeks	6MWD, FEV_1_
Yu and Jiang (15)	China	80 (45/35)	80 (43/37)	60.76 ± 6.89	60.32 ± 6.57	GOLD I- IV	Baduanjin	UC	24 weeks	6MWD
Peng et al. (12)	China	40 (16/24)	40 (18/22)	40–75	40–75	NR	Tai Chi	UC	24 weeks	6MWD, FEV_1_, FVC, CAT
Pan et al. (4)	China	42 (26/16)	42 (29/13)	60.7 ± 5.6	61.8 ± 7.2	GOLD I- IV	Baduanjin	UC	24 weeks	6MWT, FEV_1_, FVC, SGRQ
Liu (7)	China	50 (30/20)	50 (28/22)	53.5 ± 6.1	53.1 ± 5.6	NR	Tai Chi	UC	48 weeks	CAT
Liang (24)	China	41	41	60.23 ± 9.32	60.23 ± 9.32	NR	Baduanjin	UC	12 weeks	FEV_1_, FVC
Li (59)	China	43	45	51.79 ± 4.02	51.07 ± 4.49	GOLD I- II	Baduanjin	UC	24 weeks	FEV_1_, FVC, SGRQ
Jiang et al. (18)	China	40 (28/12)	40 (26/14)	57.28 ± 6.43	57.45 ± 6.39	NR	Baduanjin	UC	12 weeks	6MWT, FEV_1_, FVC, CAT
Hu et al. (10)	China	42 (30/12)	42 (25/17)	62.09 ± 3.80	62.20 ± 3.51	NR	Tai Chi	UC	12 weeks	FEV_1_, SGRQ
Guo (2017) (20)	China	30	30	45–47	45–47	NR	Baduanjin	UC	24 weeks	FEV_1_, FVC
Deng et al. (6)	China	63	63	66.6 ± 8.8	67.1 ± 9.0	NR	Tai Chi	UC	24 weeks	6MWD
Deng and Chen (25)	China	31 (31/1)	30 (29/3)	66.26 ± 5.13	66.90 ± 4.63	GOLD II- IV	Baduanjin	UC	12 weeks	FEV_1_, FVC
Chen (19)	China	60 (35/25)	60 (32/28)	66.87 ± 5.37	65.83 ± 4.21	GOLD II	Baduanjin	UC	24 weeks	FEV_1_, FVC
Chen et al. (14)	China	39	39	60.52 ± 7.24	59.67 ± 6.91	GOLD I- III	Baduanjin	UC	13 weeks	FEV_1_
Chen (9)	China	15 (7/8)	15 (5/10)	59.67 ± 3.96	62.20 ± 3.30	NR	Tai Chi	UC	16 weeks	6MWD, CAT
Dai et al. (21)	China	32 (26/6)	32 (28/4)	72.00 ± 10.93	67.94 ± 10.36	GOLD II- III	Baduanjin	UC	12 weeks	6MWT, FEV_1_, FVC, CAT
Cui et al. (8)	China	30	30	47 ± 1.2		NR	Tai Chi	UC	12 weeks	FEV_1_
Chu (13)	China	44 (24/20)	44 (25/19)	55.16 ± 7.30	54.67 ± 7.51	GOLD I- IV	Baduanjin	UC	24 weeks	6MWD
Chi et al. (11)	China	108 (96/12)	108 (94/14)	68.03 ± 6.58	67.43 ± 7.34	NR	Tai Chi	UC	52 weeks	FEV_1_, FVC, SGRQ
Pan et al. (60)	China	20 (14/6)	21 (14/7)	45–75	45–75	NR	Tai Chi	UC	8 weeks	6MWD, FEV_1_, FVC, SGRQ, CAT
Yang et al. (3)	China	44	46	63.70 ± 5.69	64.49 ± 6.10	NR	Yoga	UC	12 weeks	FEV_1_, 6MWD
Malik et al. (44)	India	30 (28/2)	30 (27/3)	61.2 ± 7.1	61.0 ± 8.2	NR	Yoga	UC	12 weeks	6MWD, CAT, SGRQ
Özdemir et al. (41)	Turkey	40 (24/16)	40 (27/13)	62.72 ± 12.13	67.12 ± 10.90	GOLD II- IV	Composite digital interventions	UC	12 weeks	CAT
Thokchom et al. (48)	India	21 (16/5)	20 (16/4)	57.80 ± 2.68	60.65 ± 1.84	GOLD I- III	Yoga	UC	12 weeks	6MWD, FEV_1_, FVC

UC, usual care, consisting of standard pharmacotherapy and health education according to GOLD guidelines, without any additional pulmonary rehabilitation; M/F: male/female; E, experimental group; C, control group; 6MWD, 6-Min Walk Distance; FEV_1_, Forced Expiratory Volume in 1 sec; FVC, Forced Vital Capacity; SGRQ, St. George's Respiratory Questionnaire; CAT, COPD Assessment Test.

### 3.3 Results of the risk of bias assessment

The risk of bias of the 47 included RCTs was assessed using the Cochrane Risk of Bias tool (Supplementary Figures S2, S3). Overall, the methodological quality of the included studies was acceptable. Most studies were adequate in terms of random sequence generation and had a low risk of bias regarding incomplete outcome data and selective reporting. The risk of bias regarding allocation concealment was low or unclear in most studies. However, the included studies also had some methodological limitations. Due to the inherent characteristics of non-pharmacological rehabilitation interventions (such as Synchronous telerehabilitation, Tai Chi, Baduanjin, and Yoga), it is often difficult to achieve blinding of participants and interventionists. Consequently, approximately half of the included studies (21 out of 47) were rated as having a high risk of bias regarding participant and staff blinding, and a similar proportion (14 out of 47) showed a high risk of bias regarding outcome assessment blinding. Because several outcomes evaluated in this study, including 6MWD, SGRQ, and CAT, are partly influenced by patient motivation, effort level, or subjective perception, the lack of blinding may have introduced potential performance and detection bias. Therefore, the observed treatment effects, particularly for patient-reported outcomes, should be interpreted with caution.

With regard to other assessment dimensions, most studies had a low risk of bias in terms of selective reporting and completeness of outcome data. A small number of studies (2 out of 47) were overall assessed as having a high risk of bias, primarily due to inadequate reporting of allocation concealment and blinding procedures. No studies were excluded solely because of a high risk of bias. The detailed results of the risk of bias assessments for each individual study are presented in Supplementary Figure S2 (risk of bias graph) and Supplementary Figure S3 (risk of bias summary).

### 3.4 Results of pairwise and network meta-analysis

#### 3.4.1 Results of paired comparisons

After harmonizing effect directions, treatment effects were interpreted according to the clinical direction of each outcome measure.

##### 3.4.1.1 For 6MWD

When comparing each intervention with UC, Baduanjin (8 studies, MD = 55.42, 95% CrI: 23.85 to 86.88) and Yoga (5 studies, MD = 62.74, 95% CrI: 17.36 to 105.32) significantly improved exercise capacity (95% CrI did not include 0). In contrast, Composite digital interventions (1 study, MD = 6.35, 95% CrI: −82.97 to 95.66), Synchronous telerehabilitation (2 studies, MD = 42.10, 95% CrI: −18.46 to 103.00) and Tai Chi (8 studies, MD = 18.68, 95% CrI: −14.13 to 50.44) did not show statistically distinguishable improvements. All pairwise comparisons exhibited high heterogeneity (I^2^ = 87.5% to 98.8%); see Supplementary Table S2.

##### 3.4.1.2 For FEV_1_

The results of pairwise comparisons between each intervention and UC showed that Baduanjin (14 studies, MD = 0.37, 95% CrI: 0.14 to 0.63) and Yoga (4 studies, MD = 0.75, 95% CrI: 0.26 to 1.20) significantly improved FEV_1_ in patients with COPD (neither 95% CrIs included 0). In contrast, Composite digital interventions (2 studies, MD = 0.04, 95% CrI: −0.53 to 0.62), Synchronous telerehabilitation (3 studies, MD = 0.54, 95% CrI: −0.15 to 1.63), and Tai Chi (8 studies, MD = 0.09, 95% CrI: −0.20 to 0.43) did not show statistically distinguishable improvements (95% CrIs included 0). All pairwise comparisons exhibited high heterogeneity (I^2^ = 79.4%−99.5%); see Supplementary Table S2.

##### 3.4.1.3 For FVC

The results of pairwise comparisons between each intervention and UC showed: Baduanjin (13 studies, MD = 0.27, 95% CrI: 0.15 to 0.40) and Yoga (2 studies, MD = 0.44, 95% CrI: 0.11 to 0.71) significantly improved FVC in patients with COPD (neither 95% CrIs included 0). In contrast, Composite digital interventions (2 studies, MD = 0.01, 95% CrI: −0.31 to 0.33) and Tai Chi (6 studies, MD = 0.06, 95% CrI: −0.13 to 0.24) did not demonstrate statistically distinguishable improvements (95% CrIs included 0). In the pairwise comparisons, heterogeneity was 0% for the comparison of Tai Chi with UC, while moderate to high heterogeneity was observed in the remaining comparisons (I^2^ = 29.2%−83.3%); see Supplementary Table S2.

##### 3.4.1.4 For the SGRQ

Results from pairwise comparisons of each intervention with UC showed that Baduanjin (4 studies, MD = −8.45, 95% CrI: −16.77 to −0.34) and Tai Chi (5 studies, MD = −9.14, 95% CrI: −16.86 to −1.84) significantly improved quality of life in patients with COPD (95% CrI did not include 0). In contrast, Composite digital interventions (2 studies, MD = −0.77, 95% CrI: −12.13 to 10.72), Synchronous telerehabilitation (2 studies, MD = −2.18, 95% CrI: −13.96 to 9.56) and Yoga (2 studies, MD = −5.11, 95% CrI: −17.84 to 7.44) did not show statistically distinguishable improvements (95% CrIs included 0). In the pairwise comparisons, heterogeneity was 0% for the comparison of Composite digital interventions and Yoga with UC, low for the comparison of Synchronous telerehabilitation with UC (I^2^ = 10.0%), and high for the remaining comparisons (I^2^ = 76.0%−92.4%); see Supplementary Table S2.

##### 3.4.1.5 For CAT

the results of pairwise comparisons between each intervention and UC showed: Baduanjin (6 studies, MD = −2.53, 95% CrI: −3.52 to −1.44), Composite digital interventions (4 studies, MD = −1.50, 95% CrI:−3.12 to −0.32), Synchronous telerehabilitation (3 studies, MD = −2.88, 95% CrI: −4.39 to −1.52), and Tai Chi (5 studies, MD = −2.97, 95% CrI: −3.99 to −1.78) all significantly reduced CAT scores in patients with COPD (improving respiratory symptoms; 95% CrI for all did not include 0, indicating statistically distinguishable differences). In contrast, Yoga (2 studies, MD = −0.13, 95% CrI: −2.15 to 1.93) did not show a statistically distinguishable improvement compared with UC (95% CrIs included 0). In the pairwise comparisons, heterogeneity was 0% for Baduanjin and Yoga compared with UC, and low for Synchronous telerehabilitation (I^2^ = 39.5%); the remaining comparisons exhibited moderate to high heterogeneity (I^2^ = 52.8%−74.4%), as shown in Supplementary Table S2.

#### 3.4.2 Network meta-regression analysis

Network meta-regression analyses were conducted to explore potential sources of heterogeneity (Supplementary Table S3). Intervention duration was significantly associated with the treatment effect estimate for 6MWD (β = 2.08, 95% CrI: 0.41 to 3.75), suggesting that longer interventions may contribute to greater improvements in exercise capacity. No significant associations were observed between intervention duration, publication year, or sample size and the effects on FEV1 or CAT outcomes. However, only a limited number of study-level covariates could be examined in the meta-regression analysis; therefore, residual heterogeneity related to unmeasured clinical and methodological factors cannot be excluded.

#### 3.4.3 Consistency Test

Model fit and between-study heterogeneity were assessed by comparing the DIC values between the consistency model and the unrelated mean effects model. The differences in DIC values across all outcome measures were minimal: 6MWD (94.61 vs. 94.62, DIC = 0.01), FEV1 (146.26 vs. 146.28, DIC = 0.02), FVC (112.29 vs. 112.30, DIC = 0.01), SGRQ (60.50 vs. 60.43, DIC = 0.07), and CAT (73.69 vs 73.66, DIC = 0.03). All DIC values were well below the threshold of 5, indicating comparable model fit between the two models and supporting the use of the consistency model. The estimated between-study heterogeneity was low to moderate across outcomes (I^2^= 5%−47%). The transitivity assumption was considered reasonable based on the similarity of key clinical and methodological characteristics (e.g., disease severity, intervention duration, and baseline population characteristics) across included studies. These similarities support the comparability of treatments across different comparisons in the network (see Supplementary Table S4).

#### 3.4.4 Network diagram

**Figure 1:**
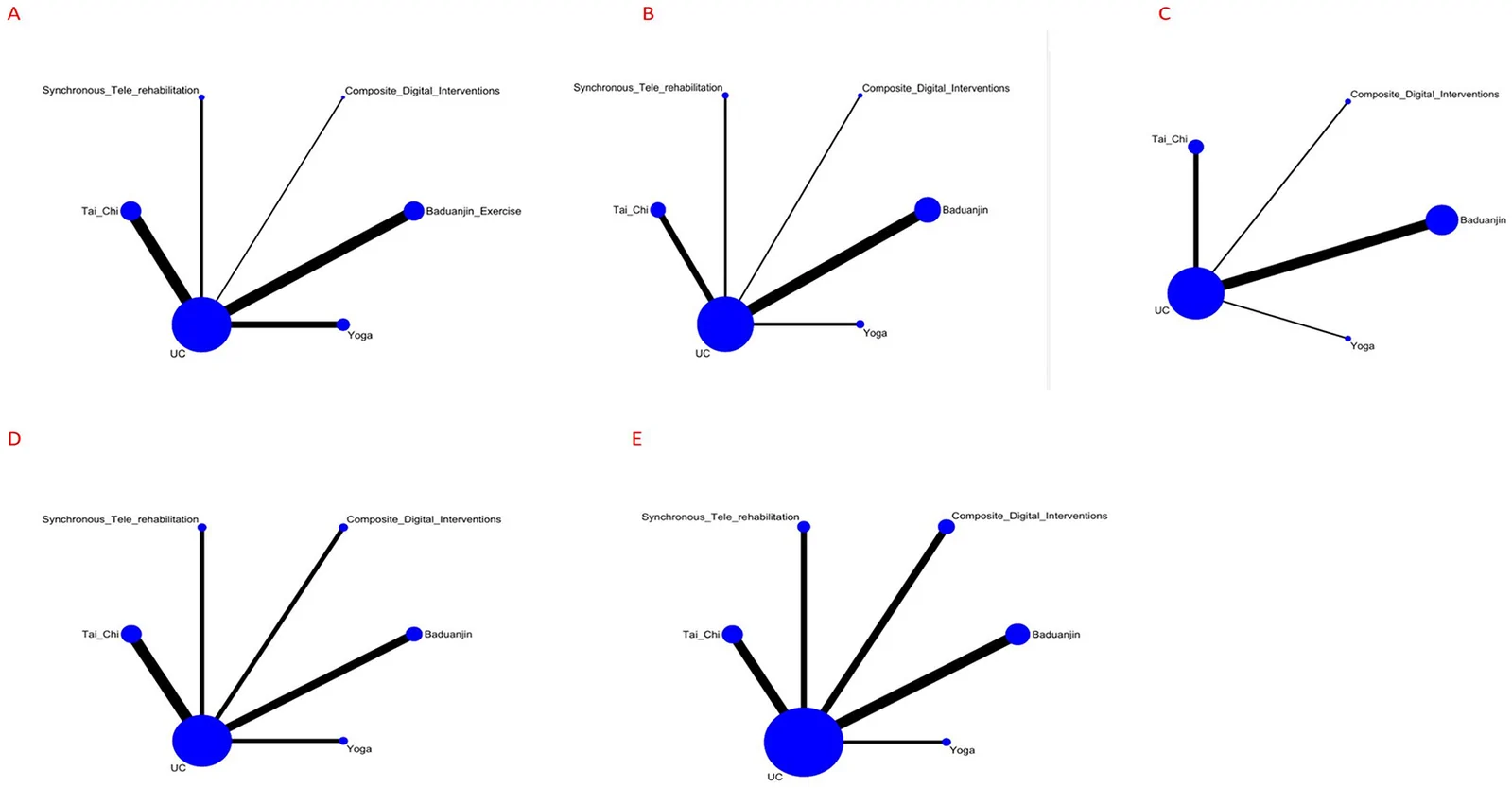
Network plots of interventions for different outcome measures. Network plots showing the available evidence for **(A)** 6MWD, **(B)** FEV1, **(C)** FVC, **(D)** SGRQ, and **(E)** CAT. The size of each node is proportional to the number of participants assigned to each intervention, while the thickness of the connecting lines represents the number of direct comparisons. UC, usual care.

This study employed a network meta-analysis and five outcome-specific treatment networks were constructed (see Figure 1) to systematically evaluate the relative rehabilitation effects of digital health interventions and traditional mind-body exercises for patients with COPD. All networks UC as a common reference node, encompassing six intervention types: UC, Synchronous telerehabilitation, Composite digital interventions, Tai Chi, Baduanjin, and Yoga. Nodes are connected by “edges”, forming an overall star-shaped network structure centered on UC. Among these, the networks for 6MWD, FEV_1_, FVC, and the CAT included all six intervention nodes, while the SGRQ network included UC, the Composite digital interventions, Tai Chi, Yoga, and Baduanjin, totaling 5 intervention nodes. All active interventions had direct comparisons with UC, whereas no direct head-to-head comparisons between active interventions were available. No isolated nodes were found in any of the comparison networks. However, the overall network exhibited a star-shaped structure centered on UC, with no closed loops. Therefore, comparisons among Yoga, Tai Chi, Baduanjin, Synchronous telerehabilitation, and Composite digital interventions were mainly based on indirect evidence through UC. Although network meta-analysis enables the estimation of relative treatment effects through indirect comparisons, the robustness of these comparisons relies on the validity of the transitivity assumption; therefore, the findings should be interpreted cautiously.

#### 3.4.5 The results of the network meta-analysis for the 6MWD outcome

Measure showed that, compared with UC, Baduanjin: MD = 55.34 (95% CrI: 23.82 to 86.65), indicating that Baduanjin was associated with greater improvement compared with UC. Yoga: MD = 62.71 (95% CrI: 17.58 to 105.19), indicating that Yoga was associated with greater improvement compared with UC. For the remaining interventions (Composite digital interventions, Synchronous telerehabilitation, and Tai Chi), the 95% CrIs included 0, and the differences were not statistically distinguishable. Comparisons among interventions: For all pairwise comparisons between Baduanjin, the Composite digital interventions, Synchronous telerehabilitation, Tai Chi, and Yoga, the 95% CrIs included 0, and the differences were not statistically distinguishable; see Supplementary Table S5.

**Figure 2:**
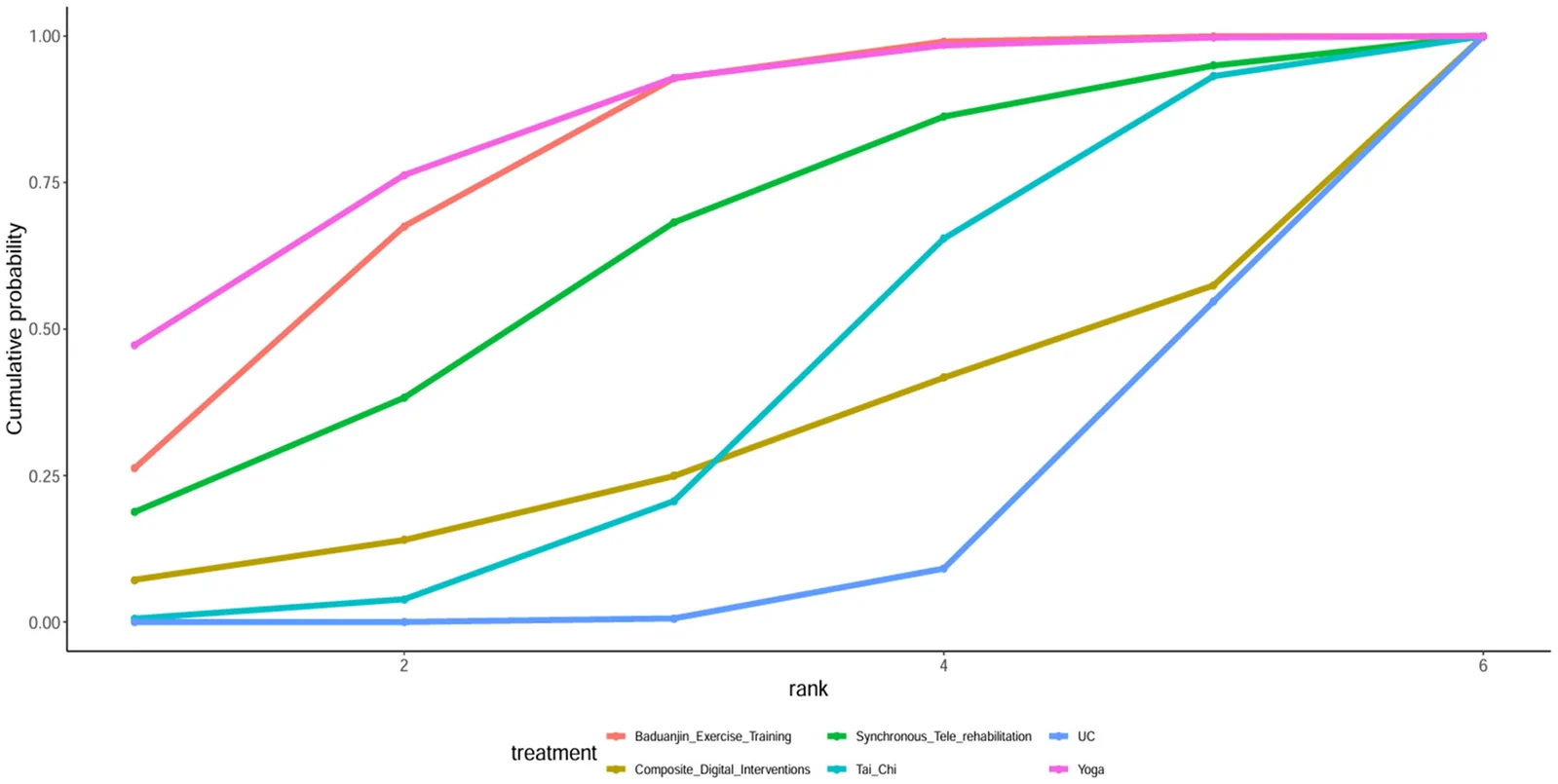
Cumulative ranking probability curves for the 6-min walk distance (6MWD). The x-axis represents the possible ranking positions (from 1 to 6), and the y-axis represents the cumulative probability that each intervention is ranked at or above a given position. Curves with higher cumulative ranking probabilities indicate a greater likelihood that the intervention is among the most effective treatments for improving 6MWD. UC, usual care.

According to the SUCRA results (Table 2 and Figure 2), using the 6MWD as the outcome measure, the probability of favorable ranking for each intervention, from highest to lowest, was as follows: Yoga (82.9%), Baduanjin (77.1%), Synchronous telerehabilitation (61.2%), Tai Chi (36.7%), Composite digital interventions (29.0%), and UC (12.8%).

**Table 2:** Cumulative frequency table.

Treatment	6MWD (%)	FEV_1_ (%)	FVC (%)	SGRQ (%)	CAT (%)
Baduanjin	77.1	64.2	74.8	76.3	69.1
Composite digital interventions	29	26.1	17.5	29.6	37.4
Synchronous telerehabilitation	61.2	73.1	NR	37.9	78.4
Tai Chi	36.7	30.6	41.0	80.7	92.3
UC	12.8	15.2	16.5	20.1	8.2
Yoga	82.9	90.5	100	55.1	14.1

6MWD, 6-Min Walk Distance; FEV_1_, Forced Expiratory Volume in 1 sec; FVC, Forced Vital Capacity; SGRQ, St. George's Respiratory Questionnaire; CAT, COPD Assessment Test; NR, not reported.

#### 3.4.6 The results of the network meta-analysis for the FEV_1_ outcome

Measure show the following comparisons of efficacy among the interventions: Comparison with UC: Baduanjin vs. UC: MD = 0.37 (95% CrI: 0.13 to 0.63), indicating that Baduanjin showed greater improvement compared with UC. Yoga vs. UC: MD = 0.75 (95% CrI: 0.26 to 1.21), indicating that Yoga was associated with greater improvement compared with UC. Comparisons of the Composite digital interventions, Synchronous telerehabilitation, and Tai Chi with UC all had 95% CrIs that included 0, indicating no statistically distinguishable differences. Comparisons between interventions: Yoga vs. Tai Chi: MD = 0.65 (95% CrI: 0.05 to 1.19), indicating a larger estimated treatment effect for Yoga than Tai Chi based on indirect evidence. Indirect comparisons between the remaining interventions all showed 95% CrIs that included 0, indicating no statistically distinguishable differences; see Supplementary Table S6.

**Figure 3:**
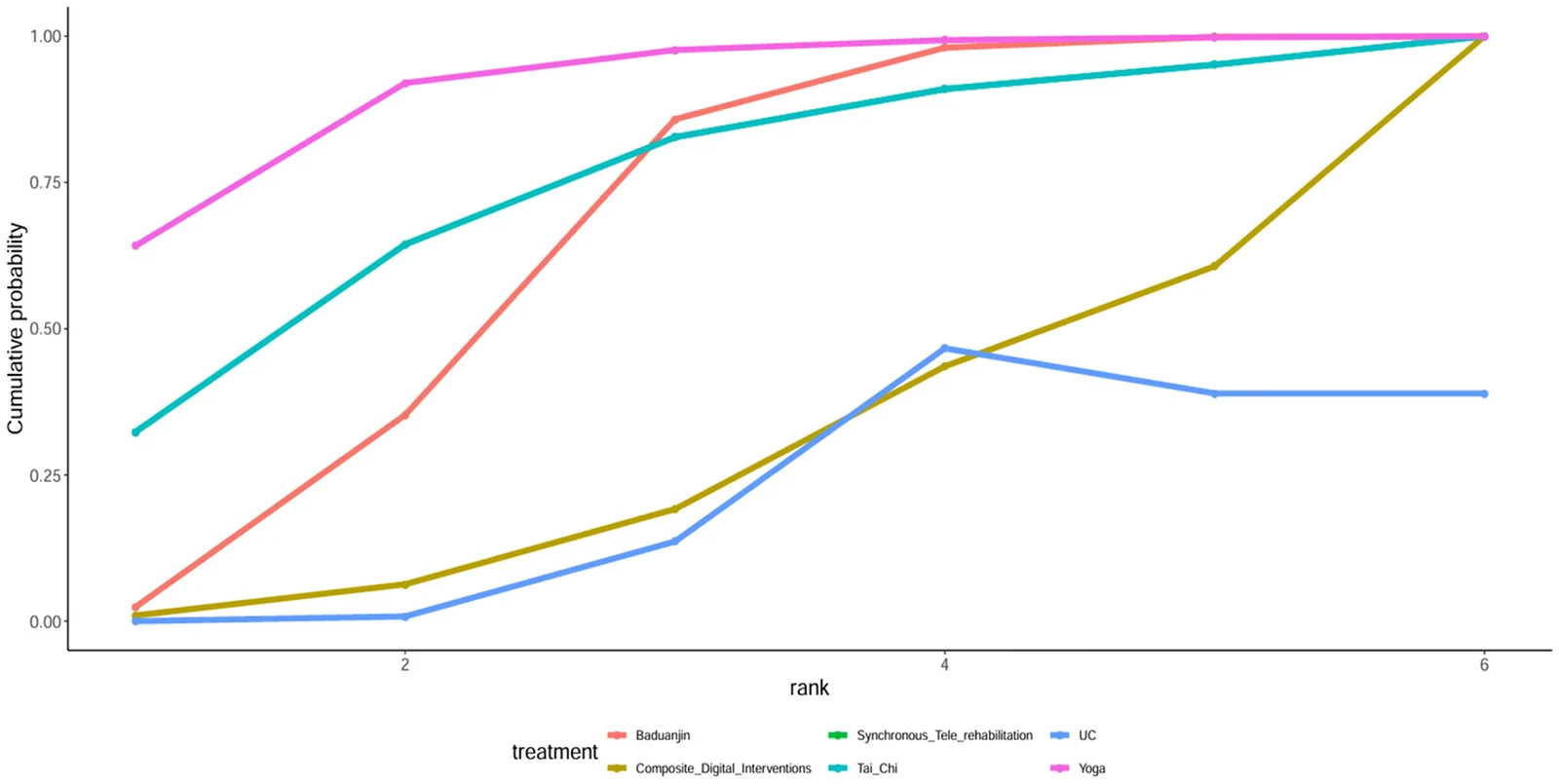
Cumulative ranking probability curves for forced expiratory volume in one second (FEV1). The x-axis represents the possible ranking positions (from 1 to 6), and the y-axis represents the cumulative probability that each intervention is ranked at or above a given position. Curves with higher cumulative ranking probabilities indicate a greater likelihood that the intervention is ranked among the most effective treatments for improving FEV1. UC, usual care.

Based on the SUCRA results (Table 2 and Figure 3), using FEV_1_ as the outcome measure, the probability of favorable ranking for each intervention,ranked from highest to lowest, was as follows: Yoga (90.5%), Synchronous telerehabilitation (73.1%), Baduanjin (64.2%), Tai Chi (30.6%), and Composite digital interventions (26.1%), UC (15.2%).

#### 3.4.7 The results of the network meta-analysis for the FVC outcome

Measure show the following comparisons of efficacy among the interventions: Comparison with UC: Baduanjin vs. UC: MD = 0.26 (95% CrI: 0.22 to 0.30), indicating that Baduanjin showed greater improvement compared with UC. Yoga vs. UC: MD = 0.51 (95% CrI: 0.43 to 0.59), indicating that Yoga showed greater improvement compared with UC. For comparisons of Composite digital interventions and Tai Chi with UC, the 95% CrIs included 0, indicating no statistically distinguishable differences.

Comparison of interventions: Baduanjin vs. Composite digital interventions: MD = 0.28 (95% CrI: 0.06 to 0.49), indicating that Baduanjin showed greater improvement compared with the Composite digital interventions. Baduanjin vs. Tai Chi: MD = 0.19 (95% CrI: 0.08 to 0.31), indicating that Baduanjin showed greater improvement compared with Tai Chi. Yoga vs. Baduanjin: MD = 0.25 (95% CrI: 0.16 to 0.34), indicating that Yoga showed a higher estimated treatment effect than Baduanjin based on indirect evidence. Yoga vs composite digital interventions: MD = 0.53 (95% CrI: 0.30 to 0.75), indicating that Yoga significantly improved compared with Composite digital interventions. Yoga vs. Tai Chi: MD = 0.44 (95% CrI: 0.31 to 0.57), indicating a larger estimated treatment effect for Yoga than Tai Chi based on indirect evidence (see Supplementary Table S7).

**Figure 4:**
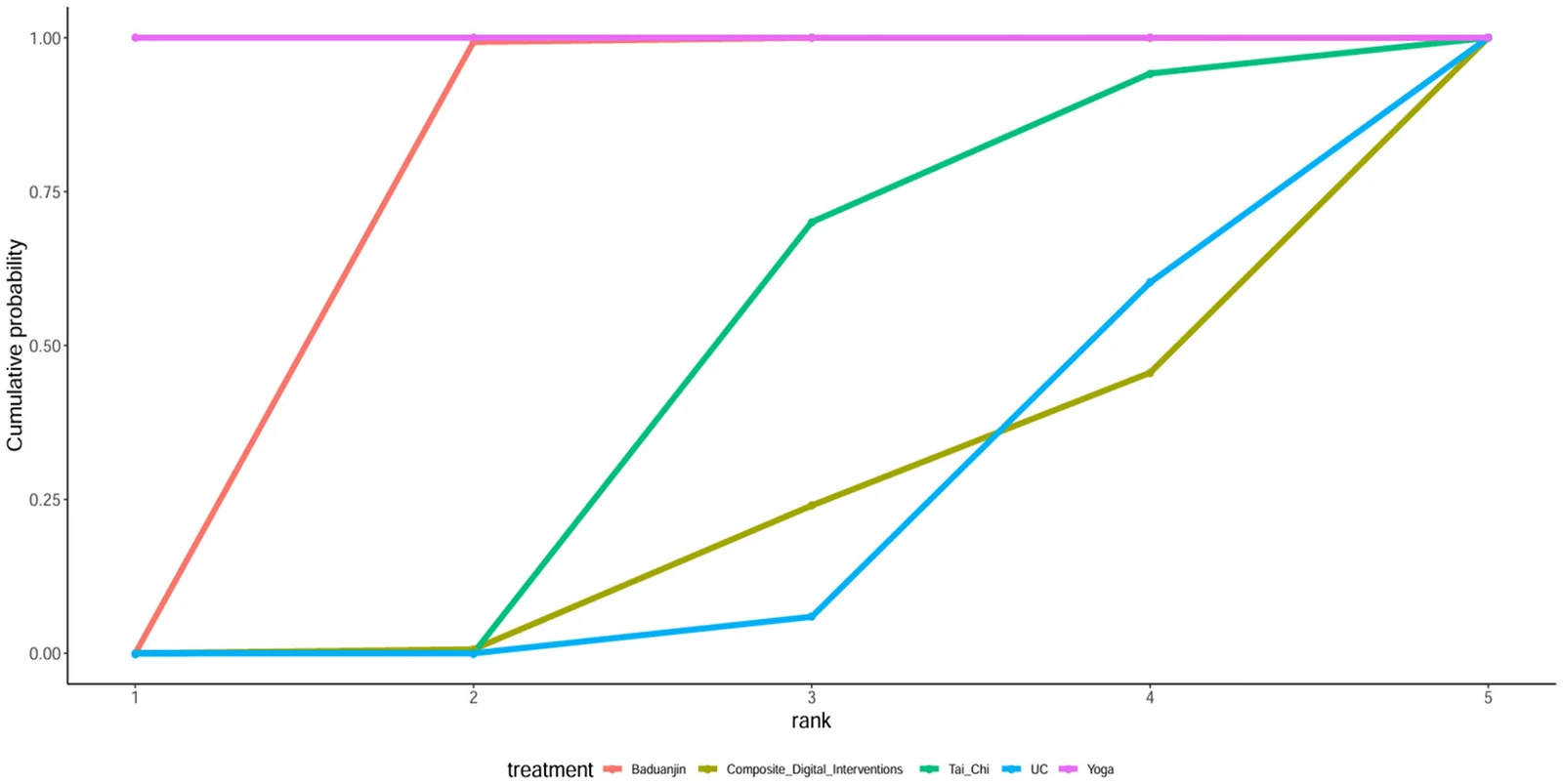
Cumulative ranking probability curves for forced vital capacity (FVC). The x-axis represents the possible ranking positions (from 1 to 5), and the y-axis represents the cumulative probability that each intervention is ranked at or above a given position. Curves with higher cumulative ranking probabilities indicate a greater likelihood that the intervention is ranked among the most effective treatments for improving FVC. UC, usual care.

Based on the SUCRA results (Table 2 and Figure 4), using FVC as the outcome measure, the probability of favorable ranking for each intervention, ranked from highest to lowest, was as follows: Yoga (100%), Baduanjin (74.8%), Tai Chi (41.0%), Composite digital interventions (17.5%) and UC (16.5%). Although Yoga demonstrated the highest SUCRA ranking for FVC, this finding should be interpreted cautiously because the FVC network included a limited number of studies and the ranking reflects relative treatment probabilities rather than absolute treatment effects. No data were available (NR) for Synchronous telerehabilitation regarding this outcome measure.

#### 3.4.8 The results of the network meta-analysis of SGRQ outcome

measures show the following comparisons of efficacy among the interventions: Comparison with UC: Baduanjin vs. UC: MD = −8.44 (95% CrI:−16.76 to −0.31), indicating that Baduanjin showed greater improvement compared with UC. Tai Chi vs. UC: MD = −9.16 (95% CrI: −16.89 to −1.76), indicating that Tai Chi showed greater improvement compared with UC. Comparisons of Composite digital interventions, Synchronous telerehabilitation, and Yoga with UC all had 95% CrIs that included 0, indicating no statistically distinguishable differences.

Comparison of interventions: For all pairwise comparisons between Baduanjin, the Composite digital interventions, Synchronous telerehabilitation, Tai Chi, and Yoga, the 95% CrIs included 0, and the differences were not statistically distinguishable (see Supplementary Table S8).

**Figure 5:**
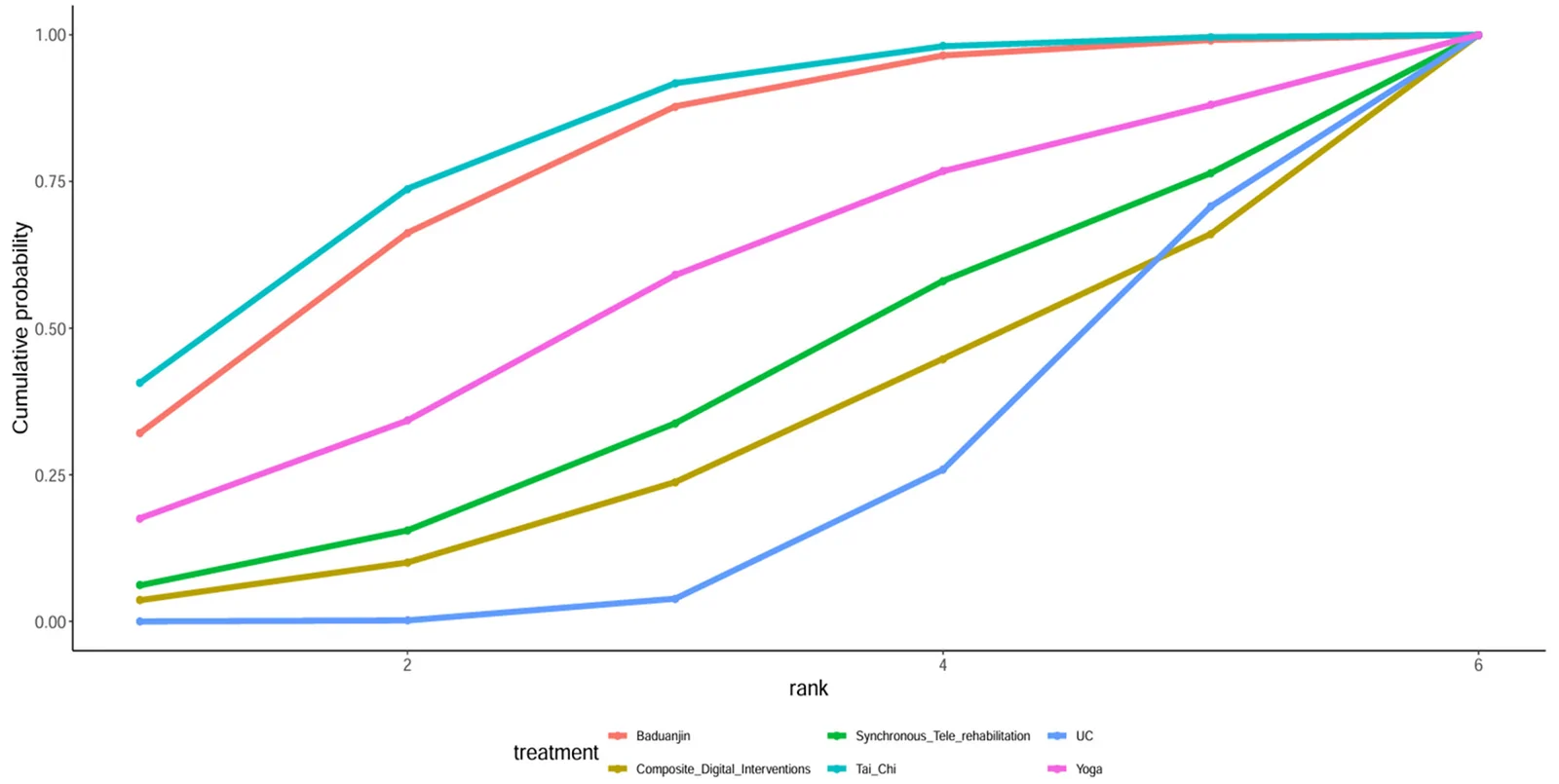
Cumulative ranking probability curves for the St. George's Respiratory Questionnaire (SGRQ). The x-axis represents the possible ranking positions (from 1 to 6), and the y-axis represents the cumulative probability that each intervention is ranked at or above a given position. Curves with higher cumulative ranking probabilities indicate a greater likelihood that the intervention is ranked among the most effective treatments for reducing SGRQ scores. UC, usual care.

Based on the SUCRA results (Table 2 and Figure 5), using the SGRQ as the outcome measure, the probability of favorable ranking for each intervention, ranked from highest to lowest, was as follows: Tai Chi (80.7%), Baduanjin (76.3%), Yoga (55.1%), Synchronous telerehabilitation (37.9%), and Composite digital interventions (29.6%), UC (20.1%).

#### 3.4.9 The results of the network meta-analysis for CAT outcome

Measures show the following comparisons of efficacy among the interventions: Comparison with UC: Baduanjin vs. UC: MD = −2.65 (95% CrI: −3.2 to −2.1), indicating that Baduanjin showed greater improvement compared with UC. Composite digital interventions vs. UC: MD = −1.11 (95% CrI:-1.84 to −0.37), indicating that Composite digital interventions showed greater improvement compared with UC. Synchronous telerehabilitation vs. UC: MD = −2.84 (95% CrI: −3.6 to −2.08), indicating that Synchronous telerehabilitation showed greater improvement compared with UC. Tai Chi vs. UC: MD = −3.15 (95% CrI: −3.75 to −2.55), indicating that Tai Chi showed greater improvement compared with UC.

Comparisons between interventions: Baduanjin vs. Composite digital interventions: MD = −1.54 (95% CrI: −2.46 to −0.62), indicating that Baduanjin showed a greater estimated improvement compared with Composite digital interventions. Baduanjin vs. Yoga: MD = −2.50 (95% CrI: −4.01, −0.98), indicating that Baduanjin showed greater improvement compared with Yoga. Synchronous telerehabilitation vs. Composite digital interventions: MD = −1.73 (95% CrI: −2.79 to −0.68), indicating that Synchronous telerehabilitation showed greater improvement compared with Composite digital interventions. Synchronous telerehabilitation vs. Yoga: MD = −2.68 (95% CrI: −4.29 to −1.09), indicating that Synchronous telerehabilitation showed greater improvement compared with Yoga. Tai Chi vs. Yoga: MD = −2.99 (95% CrI: −4.52 to −1.46), indicating that Tai Chi showed greater improvement compared with Yoga. Tai Chi vs. Composite digital interventions: MD = −2.04 (95% CrI: −2.99 to −1.09), indicating that Tai Chi showed greater improvement compared with Composite digital interventions. Indirect comparisons between the remaining interventions all showed 95% CrIs that included 0, indicating no statistically distinguishable differences; see Supplementary Table S9.

**Figure 6:**
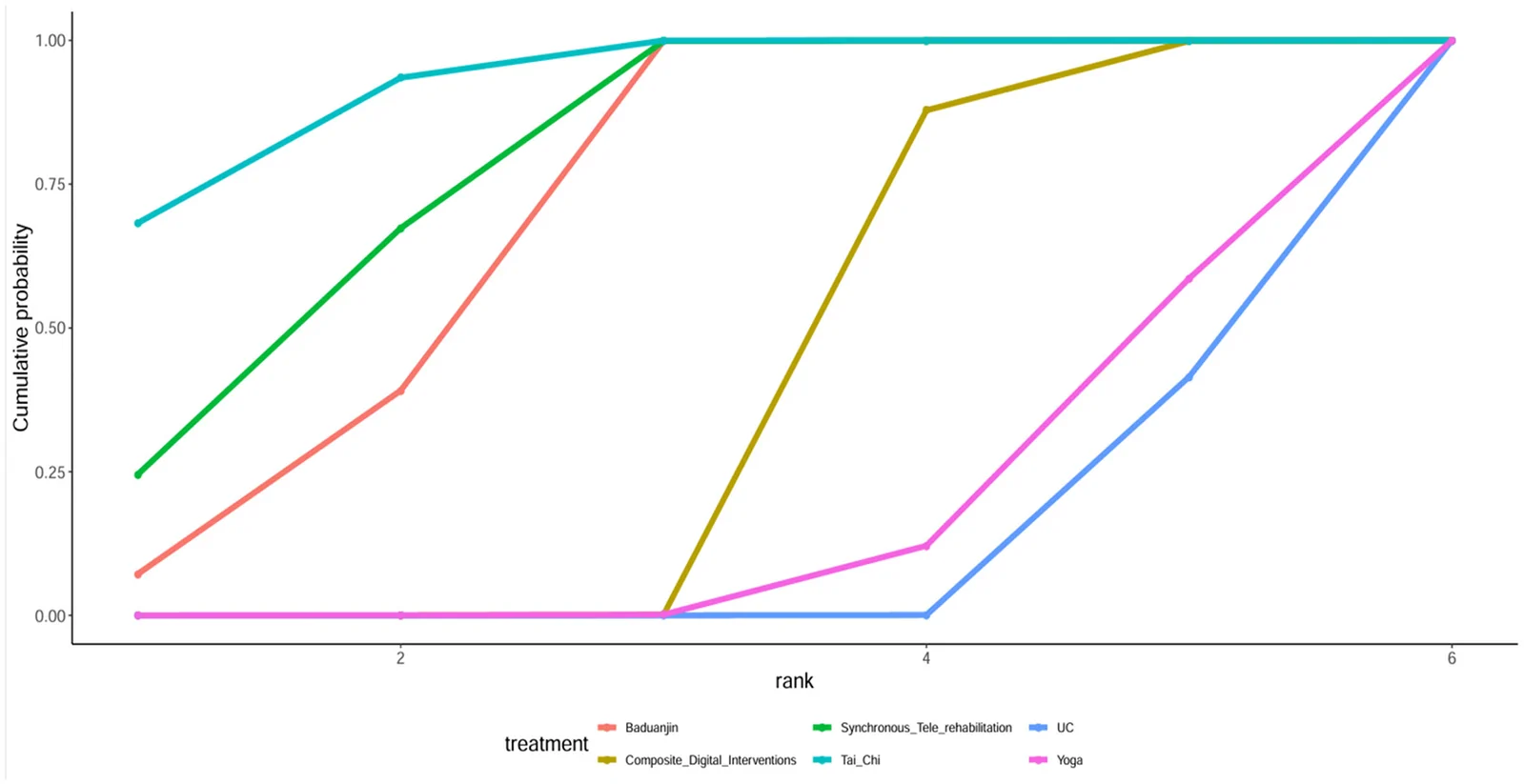
Cumulative ranking probability curves for the COPD Assessment Test (CAT). The x-axis represents the possible ranking positions (from 1 to 6), and the y-axis represents the cumulative probability that each intervention is ranked at or above a given position. Curves with higher cumulative ranking probabilities indicate a greater likelihood that the intervention is among the most effective treatments for reducing CAT scores. UC, usual care.

Based on the SUCRA results (Table 2 and Figure 6), using CAT as the outcome measure, the probability of favorable ranking for each intervention, ranked from highest to lowest, was as follows: Tai Chi (92.3%), Synchronous telerehabilitation (78.4%), Baduanjin (69.1%), Composite digital interventions (37.4%), Yoga (14.1%), UC (8.2%).

#### 3.4.10 Publication bias

Funnel plots were used to assess potential publication bias across the included outcomes (Supplementary Figures S4–S8). For most outcomes (6MWD, FEV_1_, FVC, SGRQ, and CAT), the funnel plots appeared to be approximately symmetrical, with studies distributed around the pooled effect estimates. Some degree of asymmetry was observed in a subset of outcomes. This asymmetry may be influenced by between-study heterogeneity in the included comparisons. Therefore, the possibility of publication bias cannot be completely excluded. No clear evidence of small-study effects was detected. However, given the limited number of studies in certain comparisons and the presence of heterogeneity, the interpretation of funnel plot asymmetry should be made with caution.

#### 3.4.11 GRADE assessment

The certainty of evidence for the primary outcomes was assessed using the GRADE approach (Supplementary Table S10). Overall, the certainty of evidence ranged from low to high. Moderate-certainty evidence supported the beneficial effects of Yoga and Baduanjin compared with usual care on 6MWD and FEV1, suggesting that these interventions probably improve exercise capacity and lung function. High-certainty evidence was identified for the effect of Baduanjin compared with usual care on FVC. Although Yoga showed a larger estimated effect size for FVC, the certainty of evidence was influenced by the limited number of contributing studies and should therefore be interpreted cautiously. For health-related quality of life outcomes, moderate-certainty evidence supported the effects of Tai Chi compared with usual care on SGRQ and CAT scores. However, the certainty of evidence for Baduanjin on SGRQ and CAT was rated as low, mainly due to concerns regarding risk of bias, imprecision, and potential publication bias. Across outcomes, the main reasons for downgrading the certainty of evidence were methodological limitations of the included trials, imprecision of effect estimates, and potential publication bias.

## 4 Discussion

This network meta-analysis, based on 47 randomized controlled trials involving 4,720 patients, compared the rehabilitation effects of five pulmonary rehabilitation modalities (Synchronous telerehabilitation, Composite digital interventions, Tai Chi, Baduanjin, and Yoga) in patients with stable COPD. Based on SUCRA rankings, Yoga had the highest probability of favorable ranking for exercise capacity (6MWD) and lung function outcomes (FEV1 and FVC), whereas Tai Chi had the highest probability of favorable ranking for quality-of-life outcomes (SGRQ and CAT). These rankings should be interpreted as relative probabilities rather than evidence that these interventions are definitively superior to other active rehabilitation modalities. Although several interventions showed statistically distinguishable improvements compared with UC for specific outcomes, their SUCRA rankings reflect relative probabilities of achieving better outcomes within the network rather than definitive evidence of superiority. Moreover, the evidence network was predominantly star-shaped, with UC serving as the common comparator and no direct head-to-head comparisons among active rehabilitation interventions. Consequently, the rankings among Yoga, Tai Chi, and Baduanjin were mainly derived from indirect evidence and should not be interpreted as definitive evidence of superiority. SUCRA-based rankings should therefore be considered exploratory and interpreted together with effect estimates, credible intervals, and certainty of evidence. Synchronous telerehabilitation showed potential benefits mainly for symptom improvement, particularly CAT scores. Baduanjin demonstrated consistent benefits across multiple outcomes, although the certainty of evidence varied among outcomes. However, these statistically distinguishable findings should be interpreted in conjunction with the certainty of evidence and clinical relevance, as statistical significance alone does not necessarily indicate a meaningful clinical benefit. Composite digital interventions demonstrated significant improvement in CAT scores compared with UC, although they did not show significant advantages for the remaining outcomes.

Importantly, the certainty of evidence should be considered when interpreting the treatment rankings. According to the GRADE assessment, most assessed outcomes were supported by moderate-certainty evidence, while high-certainty evidence was identified for the beneficial effect of Baduanjin compared with UC on FVC. However, the certainty of evidence for some outcomes, particularly the effects of Baduanjin on SGRQ and CAT, was rated as low due to concerns regarding risk of bias, imprecision, and potential publication bias. Therefore, treatment rankings should be interpreted alongside the certainty of evidence and clinical context rather than considered independently.

Yoga showed the highest probability of favorable ranking for improving exercise capacity (6MWD) and lung function outcomes (FEV_1_ and FVC) within the current evidence network, which is consistent with previous meta-analyses including the systematic review by Xun-Chao Liu et al. that included five RCTs (26, 61). However, this ranking reflects the probability of achieving better outcomes rather than definitive superiority over all other interventions. Therefore, the clinical interpretation of Yoga's potential benefits should consider not only the SUCRA ranking but also the magnitude of treatment effects, certainty of evidence, and clinical feasibility. The benefits of Yoga may stem from its comprehensive intervention characteristics, which integrate breath control (pranayama), physical postures (asana), and meditation (dhyana), thereby helping to improve respiratory muscle function, reduce the perception of dyspnea, and enhance exercise tolerance (53). From a clinical perspective, Yoga is particularly suitable for COPD patients whose primary concern is reduced exercise capacity, as it offers a low-cost, home-based option that requires no specialized equipment. Although Yoga showed the highest SUCRA ranking for both FEV1 and FVC, these findings should be interpreted cautiously because the FVC network included only a limited number of studies. This discrepancy may be related to differences in the physiological aspects reflected by FEV1 and FVC, variations in intervention protocols, and the limited number of studies contributing to the FVC network. Therefore, the effects of Yoga on different aspects of lung function should be interpreted separately.

Tai Chi had the highest probability of ranking favorably for improving quality of life outcomes, particularly CAT scores and SGRQ, which is consistent with several previous meta-analyses (65), including a meta-analysis by Weibing Wu et al. that included 11 RCTs and showed that Tai Chi significantly reduced SGRQ scores (62, 63). This finding was supported by moderate-certainty evidence according to the GRADE assessment. However, in this study, the improvements in 6MWD and FEV_1_ achieved by Tai Chi were not statistically distinguishable, which differs somewhat from previous studies (63). This discrepancy may be related to baseline differences in the study population and heterogeneity in the Tai Chi intervention protocols. Clinically, Tai Chi is more suitable for patients whose primary concern is impaired quality of life, and its gentle, low-intensity movements make it particularly appropriate for elderly patients with severely limited exercise tolerance. Consistent with the network meta-analysis, Tai Chi demonstrated the highest probability of improving CAT scores and significantly reduced respiratory symptom burden compared with UC. Although Synchronous telerehabilitation, Baduanjin, and Composite digital interventions also showed statistically distinguishable improvements in CAT scores compared with UC, their relatively lower SUCRA rankings indicate lower probabilities of being the best-ranked intervention within the network, rather than absence of clinically relevant benefits.

Baduanjin showed potential benefits across multiple outcomes, including 6MWD, FEV_1_, FVC, CAT, and SGRQ. However, the certainty of evidence differed across outcomes, with stronger evidence observed for FVC than for some patient-reported outcomes. These results are highly consistent with the meta-analysis by Shuai et al., which included 41 RCTs and confirmed that Baduanjin significantly improves lung function, exercise capacity, quality of life, and psychological status in patients with stable COPD (36). As a representative of traditional Chinese mind-body exercises, Baduanjin offers the advantages of gentle movements, low intensity, and ease of learning, making it particularly suitable for elderly COPD patients with limited exercise tolerance. Potential mechanisms may include strengthening the diaphragm and accessory respiratory muscles to improve ventilation, reducing airway resistance through deep and slow breathing training, and alleviating systemic inflammatory responses and oxidative stress levels (1).

Synchronous telerehabilitation significantly improved CAT scores compared with UC, while its effects on FEV1 did not reach statistical significance, consistent with the meta-analysis by Ya Li et al., which found that telerehabilitation was non-inferior to center-based rehabilitation in improving 6MWD and CAT scores (33, 34). The main advantage of Synchronous telerehabilitation lies in overcoming geographical barriers, thereby improving access to rehabilitation services for patients who cannot attend center-based programs. The Recent GOLD recommendations have highlighted the potential role of telemedicine in COPD management, and our findings support Synchronous telerehabilitation as an effective alternative for patients unable to participate in center-based rehabilitation (27).

Composite digital interventions showed a significant improvement in CAT scores compared with UC but did not demonstrate statistically distinguishable improvements for exercise capacity, lung function, or SGRQ (47). This category represents a heterogeneous group of digital rehabilitation strategies rather than a single standardized interventiong (65). The included studies differed in digital components, supervision intensity, and patient engagement requirements, which may have contributed to clinical heterogeneity and influenced the estimated treatment effects. Future research should focus on establishing standardized classification criteria and defining core intervention elements, such as exercise intensity, frequency, and feedback mechanisms (64).

Several methodological considerations warrant discussion. A high degree of heterogeneity was observed in the pairwise comparisons, which may be attributed to differences in intervention protocols (e.g., training frequency, duration per session, and total number of sessions), patient characteristics (e.g., GOLD classification, age, baseline lung function), control measures (variations in UC definitions), and outcome measurement methods (subjective scales such as SGRQ and CAT may be influenced by cultural and linguistic factors) (1, 51, 53). Although UC was used as the common comparator in this network, its specific components varied across studies, ranging from health education and medication management to routine nursing care, breathing exercises, and physical activity recommendations. This variability may introduce clinical heterogeneity and potentially influence the transitivity assumption. Nevertheless, all UC groups represented routine COPD management without the investigated rehabilitation interventions, and the similarity of baseline characteristics and study designs across comparisons supported the feasibility of network synthesis.

To further explore potential sources of heterogeneity, network meta-regression analyses were conducted. The results showed that intervention duration was significantly associated with the treatment effect of 6MWD, indicating that longer intervention periods may be associated with greater improvements in exercise capacity. However, no significant associations were observed between intervention duration, publication year, or sample size and the effects on FEV1 or CAT outcomes. These findings suggest that intervention duration may partly explain the heterogeneity observed in exercise capacity outcomes, whereas other unmeasured clinical and methodological factors may contribute to the remaining variability.

This study has several limitations. First, the number of included studies for some comparisons was limited, which may result in insufficient statistical power and imprecise effect estimates. In particular, comparisons involving digital health interventions, including Synchronous telerehabilitation and Composite digital interventions, were supported by relatively few randomized controlled trials, resulting in sparse evidence networks. Therefore, the corresponding effect estimates and SUCRA rankings should be interpreted with particular caution, as limited evidence may reduce the robustness and certainty of comparative treatment rankings. Second, the evidence network was predominantly star-shaped, with UC serving as the common comparator and no direct head-to-head comparisons among active rehabilitation interventions. Consequently, comparisons among Yoga, Tai Chi, Baduanjin, Synchronous telerehabilitation, and Composite digital interventions relied mainly on indirect evidence through UC. Although network meta-analysis provides a framework for integrating indirect evidence, the reliability of comparative rankings depends partly on the validity of the transitivity assumption and should therefore be interpreted cautiously. Future head-to-head randomized controlled trials are needed to directly compare different rehabilitation modalities. Third, substantial heterogeneity was observed in several pairwise comparisons, particularly for 6MWD, FEV_1_, and SGRQ. Although network meta-regression analyses were conducted to explore potential sources of heterogeneity, only a limited number of study-level covariates could be examined. Therefore, residual heterogeneity related to unmeasured clinical and methodological factors (e.g., baseline disease severity, intervention intensity, and follow-up duration) may remain and should be considered when interpreting the findings. Additionally, the definition of usual care varied across included trials. Although all UC groups represented routine COPD management without the evaluated rehabilitation interventions, differences in supportive care components, such as health education, medication management, nursing support, breathing exercises, and physical activity advice, may have influenced treatment effects and introduced additional heterogeneity. Future studies should provide more standardized descriptions of usual care components to improve comparability across rehabilitation trials. Fourth, the classification of Composite digital interventions as a single treatment node represents an important limitation. Because these interventions differed substantially in digital components, supervision levels, and user engagement requirements, residual heterogeneity may remain despite the network meta-analysis framework. Future studies should investigate specific digital intervention components separately or establish standardized classification criteria. Fifth, due to the unique nature of rehabilitation interventions, blinding of participants and intervention providers is inherently difficult, which may introduce performance bias. Moreover, outcomes such as 6MWD, SGRQ, and CAT may be partially influenced by participants' motivation, expectations, or subjective perceptions. Inadequate blinding of outcome assessors may further contribute to detection bias, particularly for patient-reported outcomes. These limitations should therefore be considered when interpreting the treatment effects and rankings. Sixth, although the funnel plots did not suggest major publication bias, the observed asymmetries should be interpreted cautiously in light of the existing heterogeneity, and some comparisons included a small number of studies, limiting the diagnostic power of the funnel plot. Seventh, the intervention periods in the included studies were mostly 8–24 weeks, and there was a lack of long-term follow-up data (≥1 year), making it impossible to assess the maintenance of intervention effects.

Future research should explore several directions. Long-term follow-up studies are needed to evaluate the maintenance of intervention effects and to explore the optimal frequency and intensity for sustaining benefits. Future studies with larger sample sizes should further investigate how intervention characteristics, such as duration, intensity, and frequency, influence treatment efficacy and help identify optimal rehabilitation protocols. Health economic evaluations would assess the cost-effectiveness of different interventions to inform healthcare policy decisions. Subgroup analyses should explore differential responses among patients with different GOLD classifications, ages, genders, and comorbidities to advance precision rehabilitation. Finally, mechanistic studies using physiological and neuroimaging methods could investigate the underlying mechanisms by which mind-body exercises improve lung function and exercise capacity in COPD patients.

In conclusion, this study provides comparative evidence that may inform personalized pulmonary rehabilitation strategies in COPD. Within the current evidence network, Yoga showed the highest probability of favorable ranking for exercise capacity and lung function outcomes (FEV1 and FVC), whereas Tai Chi showed the highest probability of favorable ranking for quality-of-life outcomes. Baduanjin showed potential benefits across multiple outcomes, including exercise capacity, lung function, and quality of life, although the certainty of evidence varied across outcomes. However, these findings should be interpreted cautiously because SUCRA rankings indicate relative ranking probabilities rather than definitive treatment superiority. Treatment selection should consider clinical characteristics, rehabilitation goals, accessibility, and certainty of evidence. Synchronous telerehabilitation may represent a potential alternative for patients unable to attend center-based programs. Composite digital interventions showed potential benefits for symptom improvement but require further validation because of the limited number of available trials, sparse evidence networks, and heterogeneity in intervention components. Clinicians should select rehabilitation regimens based on patients' specific goals, and future research should include more head-to-head randomized controlled trials and long-term follow-up studies to further validate these findings.

## Data Availability

The original contributions presented in the study are included in the article/Supplementary material, further inquiries can be directed to the corresponding author.

## References

[1] Yang J Liao X Li J Chen C Zhang Y Geng D. Baduanjin exercise training for elderly chronic obstructive pulmonary disease patients with mild cognitive impairment: a feasibility clinical trial. Int J Gerontol. (2025) 19:178–84. doi: 10.6890/IJGE.202507_19(3).0009

[2] Zhu Z Chen Y. The effects of seated Baduanjin exercises on activity endurance and quality of life in patients with chronic obstructive pulmonary disease (COPD). Chin J Gerontol. (2016) 36:2265–6.

[3] Yang X Lu C Zhang L Hu M Fan S. The effect of a self-designed yoga breathing exercise on pulmonary rehabilitation and depression in patients with COPD. J Nurs Educ. (2015) 30:18–21. in Chinese.

[4] Pan M Luo J Yang S. Observation on rehabilitation exercise eight-section brocade in community patients with early metaphase chronic obstructive pulmonary disease. J Chengdu Univ Tradit Chin Med. (2016) 39:49–52. in Chinese. doi: 10.13593/j.cnki.51-1501/r.2016.03.049

[5] Yuan Q Lu H Wang Y Liu Y Yu J Tian F Li Y. Telemedicine management in stabilized respiratory rehabilitation of elderly patients with moderate-to-severe chronic obstructive pulmonary disease: a randomized controlled trial. Chin Gen Pract. (2024) 27:711–6. in Chinese.

[6] Deng Z Chen H Xu Z Jiang P Du Y Song L . The Role of Tai Chi in Pulmonary Rehabilitation for Patients with Stable Chronic Obstructive Pulmonary Disease. J Anhui Vocat Coll Health Sci. (2016) 15:18–9. in Chinese.

[7] Liu D Zhang Y. Effect of Taijiquan exercise on pulmonary function in middle-aged patients with chronic obstructive pulmonary disease in stable stage. Chin Community Doctors. (2019) 35:154-7. in Chinese.

[8] Cui C Xing J Yang Y Ge P. A study on the effects of Tai Chi exercise on lung function in middle-aged patients with stable chronic obstructive pulmonary disease. Chin J Matern Child Health Res. (2016) 27(S2):535. in Chinese.

[9] Chen Z. A study on the clinical rehabilitation intervention of Tai Chi for patients with chronic obstructive pulmonary disease (COPD) in the stable phase [master's thesis]. Beijing Sport University, Beijing (2021). in Chinese. doi: 10.26961/d.cnki.gbjtu.2021.000512

[10] Hu J Han P Sang X Hu J Wu X Mo L. Clinical efficacy of Tai Chi rehabilitation training for middle-aged and elderly patients with stable chronic obstructive pulmonary disease. Chin J Gerontol. (2020) 40:5225–7. in Chinese.

[11] Chi X Zhang X Jiang H Wang J Zhang G Li Q. Observation on the efficacy of Tai Chi rehabilitation exercises on acute exacerbations of COPD patients in moderate and severe stable stage. Geriatr Study. (2021) 2:5–8. in Chinese.

[12] Peng H Wang P Wang Y Mao W Qi C. Effect of six-movement Tai Chi rehabilitation form training on pulmonary rehabilitation of patients with chronic obstructive pulmonary disease in the stable stage. Shanghai Med Pharm J. (2020) 41:58–62. in Chinese.

[13] Chu Y. The effect of Ba Duan Jin health Qigong as an adjunctive intervention for patients with chronic obstructive pulmonary disease in the stable phase. Henan Med Res. (2021) 30:5708–11. in Chinese.

[14] Chen Y Liu S Li R Zhang X Zhou L Yang G . Effects of fitness qigong eight-Duan Jin on lung function in patients with stable chronic obstructive pulmonary disease. Mod Distance Educ Tradit Chin Med. (2015) 13:16–8. in Chinese.

[15] Yu P Jiang X. Observation on the effects of Ba Duan Jin health Qigong exercise on patients with chronic obstructive pulmonary disease in the stable phase. J Wannan Med Coll. (2019) 38:607–10. in Chinese.

[16] Zhang T Zhu T Chen R Min Y Wang Y. Application of respiratory Baduanjin in patients with chronic obstructive pulmonary disease in the stable phase and its effects on blood and Qi indicators and CAT and MCFS scores. Liaoning J Tradit Chin Med. (2025) 52:120–3. in Chinese.

[17] Zhang T Ma X Chen R Yang Y. Evaluation of the effects of regular Ba Duan Jin exercise on lung function, fatigue status, and exercise tolerance in patients with chronic obstructive pulmonary disease in the stable phase. World J Integr Chin West. Med. (2019) 14:415–8. doi: 10.13935/j.cnki.sjzx.190330. in Chinese.

[18] Jiang X Yang Y Liu T Wang B Hua W. Effects of traditional Baduanjin on pulmonary rehabilitation in patients with stable chronic obstructive pulmonary disease. Chinese Health Care. (2023) 41:62–4. in Chinese.

[19] Chen Y. Effect of Baduanjin exercise rehabilitation nursing care on the rehabilitation of patients with chronic obstructive pulmonary disease. J Liaoning Univ Tradit Chin Med. (2017) 19:213–5. in Chinese.

[20] Guo J Gao Y Xie H Fang S Chen G. The effect of Baduanjin exercises on rehabilitation outcomes in patients with chronic obstructive pulmonary disease in the stable phase. Qilu Nurs J. (2016) 22:97–8. in Chinese.

[21] Dai Q Yang Q Chen Y Huang Y. The effects of Ba Duan Jin combined with respiratory training on lung function, exercise tolerance, and symptom improvement in patients with acute-phase chronic obstructive pulmonary disease. Smart Health. (2023) 9:23–7. doi: 10.19335/j.cnki.2096-1219.2023.33.006. in Chinese.

[22] Cai Q Li J Zhang X Chen J Jing C. Clinical efficacy of Baduanjin and moderate-intensity exercise training on pulmonary rehabilitation in patients with stable chronic obstructive pulmonary disease. J Liaoning Univ Tradit Chin Med. (2020) 22:139–43. in Chinese. doi: 10.13194/j.issn.1673-842x.2020.12.032

[23] Chen J Li H Sun S Lu Z Jia G Dong X. Effects of Baduanjin exercise on exercise tolerance, psychological state, and quality of of life in patients with chronic obstructive pulmonary disease. Hebei J Tradit Chin Med. (2025)47:1517–20.

[24] Liang X. Effect of a single lifting rehabilitation exercise performed by Ba Duan Jin on the rehabilitation of patients with chronic obstructive pulmonary disease. Nurs Pract Res. (2016) 13:156–7. in Chinese.

[25] Deng YF Chen JX. The effects of Baduanjin's third part on rehabilitation in stable chronic obstructive pulmonary disease patients with lung and spleen deficiency syndrome. Chin J Nurs. (2015) 50:1458–63. in Chinese.

[26] Liu XC Pan L Hu Q Dong WP Yan JH Dong L. Effects of yoga training in patients with chronic obstructive pulmonary disease: a systematic review and meta-analysis. J Thorac Dis. (2014) 6:795–802. doi: 10.3978/j.issn.2072-1439.2014.06.05 24977005 PMC4073384

[27] Chen D Long H Zhang C Chu L Li S Chen Y. Interpretation of global strategy for the diagnosis, treatment, management and prevention of chronic obstructive pulmonary disease 2025 report. Chin Gen Pract. (2025) 28:1937–49. in Chinese. doi: 10.12114/j.issn.1007-9572.2024.0588

[28] Agustí A Celli BR Criner GJ Halpin D Anzueto A Barnes P . Global initiative for chronic obstructive lung disease 2023 report: gold executive summary. Eur Respir J. (2023) 61:2300239. doi: 10.1183/13993003.00239-2023 36858443 PMC10066569

[29] Celli B Fabbri L Criner G Martinez FJ Mannino D Vogelmeier C . Definition and nomenclature of chronic obstructive pulmonary disease: time for its revision. Am J Respir Crit Care Med. (2022) 206:1317–25. doi: 10.1164/rccm.202204-0671PP 35914087 PMC9746870

[30] de Oca MM Perez-Padilla R Celli B Aaron SD Wehrmeister FC Amaral AFS . The global burden of COPD: epidemiology and effect of prevention strategies. Lancet Respir Med. (2025) 13:709–24. doi: 10.1016/S2213-2600(24)00339-4 40684784

[31] Li X Cao X Guo M Xie M Liu X. Trends and risk factors of mortality and disability adjusted life years for chronic respiratory diseases from 1990 to 2017: systematic analysis for the Global Burden of Disease Study 2017. BMJ. (2020) 368:m234. doi: 10.1136/bmj.m234 32075787 PMC7190065

[32] Garvey C. Recent updates in chronic obstructive pulmonary disease. Postgrad Med. (2016) 128:231–8. doi: 10.1080/00325481.2016.1118352 26560514

[33] Li Y Zhang H Zhao G Li J Huang H Wang L . Comparing pulmonary telerehabilitation and center-based pulmonary rehabilitation for effectiveness and adherence in chronic obstructive pulmonary disease: systematic review and meta-analysis of randomized controlled trials. J Med Internet Res. (2026) 28:e80500. doi: 10.2196/80500 41996654 PMC13089800

[34] Aburub A Darabseh MZ Badran R Eilayyan O Shurrab AM Degens H. The effects of digital health interventions for pulmonary rehabilitation in people with COPD: a systematic review of randomized controlled trials. Medicina. (2024) 60:860. doi: 10.3390/medicina60060963 38929580 PMC11206104

[35] Guo JB Chen BL Lu YM Zhang WY Zhu ZJ Yang YJ . Tai Chi for improving cardiopulmonary function and quality of life in patients with chronic obstructive pulmonary disease: a systematic review and meta-analysis. Clin Rehabil. (2016) 30:750–64. doi: 10.1177/0269215515604903 26396162

[36] Shuai Z Xiao Q Ling Y Zhang Y Zhang Y. Efficacy of traditional Chinese exercise (Baduanjin) on patients with stable COPD: a systematic review and meta-analysis. Complement Ther Med. (2023) 75:102953. doi: 10.1016/j.ctim.2023.102953 37220858

[37] Sharma M Joshi S Banjade P Ghamande SA Surani S. Global initiative for chronic obstructive lung disease (GOLD) 2023 guidelines reviewed. Open Respir Med J. (2024) 18:e18743064279064. doi: 10.2174/0118743064279064231227070344 38660684 PMC11037508

[38] Singh D Agusti A Anzueto A Barnes PJ Bourbeau J Celli BR . Global strategy for the diagnosis, management, and prevention of chronic obstructive lung disease: the GOLD science committee report 2019. Eur Respir J. (2019) 53:1900164. doi: 10.1183/13993003.00164-2019 30846476

[39] Gloeckl R Spielmanns M Stankeviciene A Plidschun A Kroll D Jarosch I . Smartphone application-based pulmonary rehabilitation in COPD: a multicentre randomised controlled trial. Thorax. (2025) 80:209–17. doi: 10.1136/thorax-2024-221803 39706685 PMC12015043

[40] Saleh S Skeie S Grundt H. Re-admission and quality of life among patients with chronic obstructive pulmonary disease after telemedicine video nursing consultation - a randomized study. Multidiscip Respir Med. (2023) 18:918. doi: 10.4081/mrm.2023.918 37753200 PMC10519187

[41] Özdemir N Enç N Gemicioglu B A. mobile health application for controlling symptoms of chronic obstructive pulmonary disease: a randomised controlled trial. Geriatr Nurs. (2026) 70:104040. doi: 10.1016/j.gerinurse.2026.104040 41926857

[42] Moy ML Wayne PM Litrownik D Beach D Klings ES Davis RB . Long-term Exercise After Pulmonary Rehabilitation (LEAP): a pilot randomised controlled trial of Tai Chi in COPD. ERJ Open Res. (2021) 7:1900164. doi: 10.1183/23120541.00025-2021 34262967 PMC8273295

[43] Kraemer KM Litrownik D Moy ML Wayne PM Beach D Klings ES . Exploring Tai Chi Exercise and Mind-Body Breathing in Patients with COPD in a Randomized Controlled Feasibility Trial. COPD. (2021) 18:288–98. doi: 10.1080/15412555.2021.1928037 34106027 PMC8283813

[44] Malik S Dua R Krishnan AS Kumar S Kumar S Neyaz O . Exercise capacity in patients with chronic obstructive pulmonary disease treated with tele-yoga versus tele-pulmonary rehabilitation: a pilot validation study. Cureus. (2022) 14:e30994. doi: 10.7759/cureus.30994 36475207 PMC9716046

[45] Dogan ZS Kokturk N. Evaluation of patients diagnosed with chronic obstructive pulmonary disease in terms of treatment compliance and quality of life after follow-up with telemedicine. European Respire J. (2024) 64:PA2518. doi: 10.1183/13993003.congress-2024.PA2518 PMC12381996 40859183

[46] Jiang Y Liu F Guo J Sun P Chen Z Li J . Evaluating an intervention program using wechat for patients with chronic obstructive pulmonary disease: randomized controlled trial. J Med Internet Res. (2020) 22:e17089. doi: 10.2196/17089 32314971 PMC7201319

[47] Vorrink SN Kort HS Troosters T Zanen P Lammers JJ. Efficacy of an mHealth intervention to stimulate physical activity in COPD patients after pulmonary rehabilitation. Eur Respir J. (2016) 48:1019–29. doi: 10.1183/13993003.00083-2016 27587557

[48] Thokchom SK Gulati K Ray A Menon BK. Rajkumar. Effects of yogic intervention on pulmonary functions and health status in patients of COPD and the possible mechanisms. Complement Ther Clinic Pract. (2018) 33:20–6. doi: 10.1016/j.ctcp.2018.07.008 30396622

[49] Papp ME Wändell PE Lindfors P Nygren-Bonnier M. Effects of yogic exercises on functional capacity, lung function and quality of life in participants with obstructive pulmonary disease: a randomized controlled study. Eur J Phys Rehabil Med. (2017) 53:447–61. doi: 10.23736/S1973-9087.16.04374-4 27830924

[50] Kantatong T Panpanich R Deesomchok A Sungkarat S Siviroj P. Effects of the tai chi qigong programme on functional capacity, and lung function in chronic obstructive pulmonary disease patients: a ramdomised controlled trial. J Tradit Complement Med. (2020) 10:354–9. doi: 10.1016/j.jtcme.2019.03.008 32695652 PMC7365785

[51] Luo C Jiang H Li H Chi X. Effects of Tai Chi on patients with moderate to severe COPD in stable phase. Medicin. (2023) 102:e33503. doi: 10.1097/MD.0000000000033503 37026910 PMC10082252

[52] Huang X Song X Sun M Hou Y Nan J Gao J . Effectiveness of digital co-creation platform in remote pulmonary rehabilitation for older adults with chronic obstructive pulmonary disease: a randomized controlled trial. Front Public Health. (2025) 13:1708607. doi: 10.3389/fpubh.2025.1708607 41293596 PMC12640875

[53] Kaminsky DA Guntupalli KK Lippmann J Burns SM Brock MA Skelly J . Effect of yoga breathing (pranayama) on exercise tolerance in patients with chronic obstructive pulmonary disease: a randomized, controlled trial. J Altern Complement Med. (2017) 23:696–704. doi: 10.1089/acm.2017.0102 28714735 PMC5610410

[54] Køpfli ML Børgesen S Jensen MS Hyldgaard C Bell C Andersen FD. Effect of telemonitoring on quality of life for patients with chronic obstructive pulmonary disease-A randomized controlled trial. Chron Respir Dis. (2023) 20:14799731231157771. doi: 10.1177/14799731231157771 36775280 PMC9926364

[55] Tupper OD Gregersen TL Ringbaek T Brøndum E Frausing E Green A . Effect of tele-health care on quality of life in patients with severe COPD: a randomized clinical trial. Int J Chron Obstruct Pulmon Dis. (2018) 13:2657–62. doi: 10.2147/COPD.S164121 30214183 PMC6122889

[56] Zhang D Wang G Li K Zhang P. Application of Telemedicine-Based Traditional Chinese Medicine Pulmonary Rehabilitation Therapy in Patients with Moderate-to-Severe Chronic Obstructive Pulmonary Disease in the Stable Phase. J Changzhi Medical College. (2025) 39:444–8. doi: 10.21751/FRM-39-3-4-71

[57] Zhang W. A Study on the Efficacy of Telerehabilitation on Health-Related Quality of Life in Patients with Chronic Obstructive Pulmonary Disease in the Stable Phase. Inner Mongolia: Inner Mongolia Medical University (2025).

[58] Eini A. Clinical efficacy of six-form Tai Chi on pulmonary rehabilitation in 42 patients with chronic obstructive pulmonary disease in the stable phase.). Urumqi: Xinjiang Medical University (2021).

[59] Li M. The effects of Ba Duan Jin health Qigong on cognitive function, mood, and quality of life in patients with chronic obstructive pulmonary disease in the stable phase. Shenyang: Shenyang Sport University (2018).

[60] Pan Y Wang Z Min J Huang Y Xiao W Mao B . Evaluation of the Efficacy of the 24-Form Simplified Tai Chi in Pulmonary Rehabilitation During the Stable Phase of Chronic Obstructive Pulmonary Disease. Proceedings of the Second Academic Symposium of the Respiratory Disease Expert Committee, Branch of Integrated Traditional Chinese and Western Medicine Physicians, Chinese Medical Doctor Association; Chengdu (2017). p. 156.

[61] Zhu Y Zhang Z Du Z Zhai F. Mind-body exercise for patients with stable COPD on lung function and exercise capacity: a systematic review and meta-analysis of RCTs. Sci Rep. (2024) 14:18300. doi: 10.1038/s41598-024-69394-4 39112599 PMC11306772

[62] Wu W Liu X Wang L Wang Z Hu J Yan J. Effects of Tai Chi on exercise capacity and health-related quality of life in patients with chronic obstructive pulmonary disease: a systematic review and meta-analysis. Int J Chron Obstruct Pulmon Dis. (2014) 9:1253–63. doi: 10.2147/COPD.S70862 25404855 PMC4230171

[63] Yang Y Yang L Yang X Tian Y. Effects of Tai Chi on Lung Function, Exercise Capacity and Psychosocial Outcomes in Patients With Chronic Obstructive Pulmonary Disease: Systematic Review and Meta-analysis of Randomized Controlled Trials. Biol Res Nurs. (2023) 25:635–46. doi: 10.1177/10998004231178318 37210672

[64] Schoeppe S Alley S Van Lippevelde W Bray NA Williams SL Duncan MJ . Efficacy of interventions that use apps to improve diet, physical activity and sedentary behaviour: a systematic review. Int J Behav Nutr Phys Act. (2016) 13:127. doi: 10.1186/s12966-016-0454-y 27927218 PMC5142356

[65] Zhang H Hu D Xu Y Wu L Lou L. Effect of pulmonary rehabilitation in patients with chronic obstructive pulmonary disease: a systematic review and meta-analysis of randomized controlled trials. Ann Med. (2022) 54:262–73. doi: 10.1080/07853890.2021.1999494 35037535 PMC8765243

