# Supplementary material for: Comparative efficacy of digital health interventions and traditional mind-body exercises for pulmonary rehabilitation in chronic obstructive pulmonary disease: a systematic review and network meta-analysis
Source: Front Med (Lausanne). 2026 Aug 24;13:1871103. doi: 10.3389/fmed.2026.1871103 (PMC13564113; doi:10.3389/fmed.2026.1871103)
Supplement: Supplementary file 1 [file Data_Sheet_1.docx]

Figure S1 Flowchart of Literature Search

Records removed *before screening*:

Duplicate records removed (n = 4893)

Records marked as ineligible by automation tools (n =809)

Records removed for other reasons (n =368)

Records identified from: Databases (n = 10468)

PubMed (n=2982)

Embase (n=1648)

Cochrane library (n=2614)

Web of science (n=1552)

CNKI (n=514)

Wang fang (n=803)

VIP (n=355)

**Identification**

Records screened

(n =4398)

Records excluded by Reading the title and abstract (n =4088)

Reports sought for retrieval

(n = 310)

Reports not retrieved

(n = 0)

**Screening**

Reports assessed for eligibility

(n = 310)

Reports excluded:

There was no relevant

outcome (n=45)

Data not available (n =10)

Inappropriate Intervention

(n=109）

Full text not available(n=57）

Non - RCT(n=42)

Reports of included studies

(n = 47)

**Included**

Figure S2 risk of bias graph

Figure S3 risk of bias summary

*Green = low risk, yellow = unclear risk, red = high risk.

Figure S4 6MWD Funnel Chart

Figure S5	FEV_1_ Funnel Chart

Figure S6	FVC Funnel Chart

Figure S7 SGRQ Funnel Chart

Figure S 8 CAT Funnel Char

Table S1 search strategy

**PubMed**

(((((("Pulmonary Disease, Chronic Obstructive"[Mesh]) OR (COPD[Title/Abstract])) OR (Chronic Obstructive Pulmonary Disease[Title/Abstract])) OR (Chronic Obstructive Lung Disease[Title/Abstract])) OR (Emphysema[Title/Abstract])) OR (Chronic Bronchitis[Title/Abstract])) AND ((((((((((((((((((((("Telerehabilitation"[Mesh]) OR (Tele-rehabilitation[Title/Abstract])) OR (Telehealth[Title/Abstract])) OR (Telemedicine[Title/Abstract])) OR (Telemonitoring[Title/Abstract])) OR (Remote Monitoring[Title/Abstract])) OR (Digital Health[Title/Abstract])) OR (Mobile Health[Title/Abstract])) OR (mHealth[Title/Abstract])) OR (eHealth[Title/Abstract])) OR (App[Title/Abstract])) OR (Apps[Title/Abstract])) OR (Smartphone[Title/Abstract])) OR (Smartphone-based[Title/Abstract])) OR (Wearable[Title/Abstract])) OR (Activity Tracker[Title/Abstract])) OR (Web-based[Title/Abstract])) OR (Internet-based[Title/Abstract])) OR (Online[Title/Abstract])) OR (Remote Support[Title/Abstract])) OR ((((((((((("Tai Ji"[Mesh]) OR ("Yoga"[Mesh])) OR (Tai Chi[Title/Abstract])) OR (taiji[Title/Abstract])) OR (qigong[Title/Abstract])) OR (baduanjin[Title/Abstract])) OR (yoga[Title/Abstract])) OR (mind-body[Title/Abstract])) OR (mind body[Title/Abstract])) OR (breathing exercise[Title/Abstract])) OR (respiratory muscle training[Title/Abstract])))

**Web of Science**

TS=("chronic obstructive pulmonary disease" OR COPD OR "chronic obstructive lung disease" OR emphysema OR "chronic bronchitis") AND TS=("telerehabilitation" OR "tele-rehabilitation" OR telehealth OR telemedicine OR telemonitoring OR "remote monitoring" OR "digital health" OR "mobile health" OR mhealth OR ehealth OR app OR apps OR smartphone OR "smartphone-based" OR wearable OR "activity tracker" OR "web-based" OR "internet-based" OR online OR "remote support" OR "Tai Chi" OR taiji OR qigong OR baduanjin OR yoga OR "mind-body" OR "breathing exercise" OR "respiratory muscle training") AND TS=("randomized controlled trial" OR "randomised controlled trial" OR RCT*)

**Cochrane Library**

#1	MeSH descriptor: [Pulmonary Disease, Chronic Obstructive] explode all trees	8381

#2	(COPD):ti,ab,kw OR (Chronic Obstructive Pulmonary Disease):ti,ab,kw OR (Chronic Obstructive Lung Disease):ti,ab,kw OR (Emphysema):ti,ab,kw OR (Chronic Bronchitis):ti,ab,kw	29246

#3	#1 OR #2	29246

#4	Telerehabilitation	2329

#5	(Tele-rehabilitation):ti,ab,kw OR (Telehealth):ti,ab,kw OR (Telemedicine):ti,ab,kw OR (Telemonitoring):ti,ab,kw OR (Remote Monitoring):ti,ab,kw	15675

#6	(Mobile Health):ti,ab,kw OR (mHealth):ti,ab,kw OR (eHealth):ti,ab,kw OR (App):ti,ab,kw OR (Apps):ti,ab,kw	25864

#7	#4 OR #5 OR #6	38714

#8	Tai Ji	790

#9	(Yoga):ti,ab,kw OR ("Tai Chi Chuan"):ti,ab,kw OR (taiji):ti,ab,kw OR (qigong):ti,ab,kw OR (baduanjin):ti,ab,kw 8830

#10	(yoga):ti,ab,kw OR (mind-body):ti,ab,kw OR (mind body):ti,ab,kw OR (breathing exercise):ti,ab,kw OR (respiratory muscle training):ti,ab,kw	19052

#11	#8 OR #9 OR #10	20808

#12	#7 OR #11	58743

#13	#3 AND #12	2614

**Embase**

#1	'chronic obstructive lung disease'/exp OR 'chronic obstructive lung disease' OR copd:ti,ab,kw OR 'chronic obstructive pulmonary disease':ti,ab,kw OR 'chronic obstructive lung disease':ti,ab,kw OR emphysema:ti,ab,kw OR 'chronic bronchitis':ti,ab,kw	298,319

#2	'chronic obstructive lung disease'/exp OR 'chronic obstructive lung disease' OR copd:ti,ab,kw OR 'chronic obstructive pulmonary disease':ti,ab,kw OR 'chronic obstructive lung disease':ti,ab,kw OR emphysema.ti,ab,kw OR 'chronic bronchitis':ti,ab,kw	298,319

#3	'pulmonary disease, chronic obstructive'/exp OR' pulmonary disease, chronic obstructive' OR (pulmonary AND (disease,'/exp OR disease,) AND chronic AND obstructive) OR 'copd' /exp OR copd OR 'chronic obstructive pulmonary disease'/exp OR 'chronic obstructive pulmonary disease' OR (chronic AND obstructive AND pulmonary AND ('disease'/exp OR disease)) OR 'chronic obstructive lung disease'/exp OR 'chronic obstructive lung disease' OR (chronic AND obstructive AND ('lung'/exp OR lung) AND (disease '/exp OR disease)) OR 'emphysema'/exp OR emphysema OR 'chronic bronchitis'/exp OR 'chronic bronchitis' OR (chronic AND ('bronchitis'/exp OR bronchitis))	357,350

#4	'telerehabilitation' /exp OR telerehabilitaton OR 'tele rehabilitation'/exp OR 'tele rehabilitation' OR 'telehealth' /exp OR telehealth OR telemedicine'/exp OR telemedicine OR 'telemonitoring'/exp OR telemonitoring OR 'remote monitoring'/exp OR 'remote monitoring' OR (remote AND ('monitoring'/exp OR monitoring)) OR 'digital health'/exp OR 'digital health' OR (digital AND ('health'/exp OR health)) OR 'mobile health'/exp OR 'mobile health' OR ( mobile AND (health'/exp OR health) OR 'mhealth'/exp OR mhealth OR 'ehealth'/exp OR ehealth OR 'app'/exp OR app OR apps OR 'smartphone'/exp OR smartphone OR 'smartphone based' OR wearable OR 'activity tracker'/exp OR 'activity tracker' OR (('activity' /exp OR activity) AND ('tracker'/exp OR tracker)) OR 'web based' OR 'internet based' OR 'online'/exp OR online OR 'remote support' OR (remote AND ('support'/exp OR support) OR 'tai ji'/exp OR 'tai ji' OR (tai AND ji) OR 'Tai Chi'/exp OR 'Tai Chi' OR (tai AND chi) OR taiji OR 'qigong'/exp OR qigong OR 'baduanjin'/exp OR baduanjin OR 'yoga'/exp OR yoga OR 'mind body' OR('mind'/exp OR mind) AND ('body'/exp OR body) OR 'breathing exercise' /exp OR 'breathing exercise' OR ('breathing'/exp OR breathing) AND ('exercise'/exp OR exercise)) OR 'respiratory muscle training'/exp OR 'respiratory muscle training' OR (('respiratory'/exp OR respiratory) AND ('muscle'/exp OR muscle) AND ('training' /exp OR training))	1,555,196

#5	#3 AND #4	21,961

#6	'randomized controlled trials as topic'/exp OR 'randomized controlled trials as topic' OR (randomized AND controlled AND trials AND as AND topic) OR rct OR random	945,819

#7	#3 AND #4 AND #6	1,648

**CNKI**

(Subject: Lung diseases, chronic obstructive + COPD + chronic obstructive pulmonary disease + emphysema + chronic bronchitis) AND (Subject: Telerehabilitation + digital health + mobile health + smartphones + wearable devices + Tai Chi + Qigong + Baduanjin + yoga)

**VIP**

((((((Title or Keywords=Lung Disease, Chronic Obstructive OR Title or Keywords=COPD) OR Title or Keywords = Chronic Obstructive Pulmonary Disease) OR Title or Keywords = Chronic Obstructive Pulmonary Disease) OR Title or Keywords = Emphysema) OR Title or Keywords = Chronic Bronchitis) AND ((((((( ((Title or Keywords = Telerehabilitation OR Title or Keywords = Digital Health) OR Title or Keywords = Mobile Health) OR Title or Keywords = Smartphone) OR Title or Keywords = Wearable Devices) OR Title or Keywords = Tai Chi) OR Title or Keyword = Tai Chi) OR Title or Keyword = Qigong) OR Title or Keyword = Baduanjin) OR Title or Keyword = Yoga))

**Wang fang**

Subject: (Lung disease, Chronic obstructive OR COPD OR Chronic obstructive pulmonary disease OR Chronic obstructive pulmonary disease OR Emphysema OR Chronic bronchitis) and Subject: (Telerehabilitation OR Digital health OR Mobile health OR Smartphones OR Wearable devices OR Tai Chi OR Tai Chi Chuan OR Qigong OR Baduanjin OR Yoga)

Table S2 Pairwise comparison of meta-analysis results

| **Outcomes** | **Pairwise meta-analysis** | **No of study** | **Heterogeneity (%)** | **MD (95% CrI)** |
| --- | --- | --- | --- | --- |
| 6MWD | Baduanjin vs UC | 8 | 91.0 | 55.41 (23.87, 86.87) |
|  | Composite digital interventions vs UC | 1 | 0 | 6.35 (-82.97, 95.66) |
|  | Synchronous telerehabilitation vs UC | 2 | 98.7 | 42.10 (-18.46, 103.00) |
|  | Tai Chi vs UC | 8 | 87.5 | 18.68 (-14.13, 50.44) |
|  | Yoga vs Uc | 5 | 98.8 | 62.74 (17.36, 105.32) |
| FEV_1_ | Baduanjin vs UC | 14 | 82.9 | 0.37 (0.14, 0.63) |
|  | Composite digital interventions vs UC | 2 | 79.4 | 0.04 (-0.53, 0.62) |
|  | Synchronous telerehabilitation vs UC | 3 | 97.3 | 0.54 (-0.15, 1.63) |
|  | Tai Chi vs UC | 8 | 84.8 | 0.09 (-0.20, 0.43) |
|  | Yoga vs Uc | 4 | 99.5 | 0.75 (0.26, 1.20) |
| FVC | Baduanjin vs UC | 13 | 76.3 | 0.27 (0.15, 0.40) |
|  | Composite digital interventions vs UC | 2 | 29.2 | 0.01 (-0.31, 0.33) |
|  | Tai Chi vs UC | 6 | 0 | 0.06 (-0.13, 0.24) |
|  | Yoga vs UC | 2 | 83.3 | 0.44 (0.11, 0.71) |
| SGRQ | Baduanjin vs UC | 4 | 76.0 | -8.45 (-16.77, -0.34) |
|  | Composite digital interventions vs UC | 2 | 0 | -0.77 (-12.13, 10.72) |
|  | Synchronous telerehabilitation vs UC | 2 | 10.0 | -2.18 (-13.96, 9.56) |
|  | Tai Chi vs UC | 5 | 92.4 | -9.14 (-16.86, -1.84) |
|  | Yoga vs UC | 2 | 0 | -5.11 (-17.84, 7.44) |
| CAT | Baduanjin vs UC | 6 | 0 | -2.53 (-3.52, -1.44) |
|  | Composite digital interventions vs UC | 4 | 74.43 | -1.50 (-3.12, -0.32) |
|  | Synchronous telerehabilitation vs UC | 3 | 39.5 | -2.88 (-4.39, -1.52) |
|  | Tai Chi vs UC | 5 | 52.8 | -2.97 (-3.99, -1.78) |
|  | Yoga vs UC | 2 | 0 | -0.13 (-2.15, 1.93) |

*MD values were oriented according to the clinical interpretation of each outcome. Positive values indicate improvement for 6MWD, FEV₁, and FVC, whereas negative values indicate improvement for SGRQ and CAT.

**6MWD**

FEV_1_

FVC

SGRQ

CAT

Table S3 Network meta-regression analysis

| **Outcome** | **Covariate** | **No. of studies** | **β coefficient** | **95% CI** | **P value** |
| --- | --- | --- | --- | --- | --- |
| 6MWD | Intervention duration | 25 | 2.08 | 0.41 to 3.75 | 0.018 |
|  | Publication year | 25 | 0.34 | −0.50 to 1.18 | 0.426 |
|  | Sample size | 25 | 0.02 | −0.05 to 0.09 | 0.538 |
| FEV₁ | Intervention duration | 31 | 0.006 | −0.003 to 0.015 | 0.176 |
|  | Publication year | 31 | -0.002 | −0.009 to 0.005 | 0.571 |
|  | Sample size | 31 | 0.0004 | −0.0008 to 0.0015 | 0.494 |
| CAT | Intervention duration | 20 | -0.12 | −0.35 to 0.10 | 0.281 |
|  | Publication year | 20 | 0.05 | −0.15 to 0.24 | 0.612 |
|  | Sample size | 20 | 0.01 | −0.04 to 0.06 | 0.748 |

Table S4 Consistent Modeling

| **Outcomes** | **Consistency test** | **Inconsistency test** | **I2(%)** |
| --- | --- | --- | --- |
| 6MWD | 94.61 | 94.62 | 5 |
| FEV_1_ | 146.26 | 146.28 | 34 |
| FVC | 112.29 | 112.30 | 47 |
| SGRQ | 60.50 | 60.43 | 7 |
| CAT | 73.69 | 73.66 | 20 |

Table S5 6MWD League Schedule

| **MD (95%CrI)** | | | | |  |
| --- | --- | --- | --- | --- | --- |
| Baduanjin |  |  |  |  |  |
| 49.39 (-45.32, 143.1) | Composite digital interventions |  |  |  |  |
| 13.23 (-55.66, 81.71) | -36.2 (-143.43, 71.69) | Synchronous telerehabilitation |  |  |  |
| 36.56 (-8.02, 81.66) | -12.73 (-106.11, 81.58) | 23.2 (-44.48, 92.48) | Tai_Chi |  |  |
| 55.34 (23.82, 86.65)* | 6.35 (-82.38, 95.00) | 42.01 (-18.49, 103.11) | 18.77 (-13.49, 50.28) | UC |  |
| -7.34 (-60.08, 47.75) | -56.55 (-153.67, 44.08) | -20.66 (-94, 56.26) | -43.88 (-97.06, 11.41) | 62.71 (17.58, 105.19)* | Yoga |

*Means P < 0.05.

TableS6 FEV_1_ League Table

| **MD (95%CrI)** | | | | | |
| --- | --- | --- | --- | --- | --- |
| Baduanjin |  |  |  |  |  |
| 0.33 (-0.29, 0.97) | Composite digital interventions |  |  |  |  |
| -0.16 (-1.26, 0.57) | -0.49 (-1.75, 0.37) | Synchronous telerehabilitation |  |  |  |
| 0.28 (-0.12, 0.66) | -0.05 (-0.73, 0.58) | 0.44 (-0.3, 1.54) | Tai_Chi |  |  |
| 0.37 (0.13, 0.63)* | 0.04 (-0.53, 0.62) | 0.53 (-0.16, 1.62) | 0.09 (-0.2, 0.42) | UC |  |
| -0.37 (-0.89, 0.18) | -0.7 (-1.43, 0.05) | -0.21 (-1.03, 1.01) | 0.65 (0.05,1.19)* | 0.75 (0.26,1.21)* | Yoga |

*Means P < 0.05.

TableS7 FVC Schedule

| **MD (95%CrI)** | | | | |
| --- | --- | --- | --- | --- |
| Baduanjin |  |  |  |  |
| 0.28 (0.06, 0.49)* | Composite digital interventions |  |  |  |
| 0.19 (0.08, 0.31)* | -0.08 (-0.32, 0.16) | Tai_Chi |  |  |
| 0.26 (0.22, 0.30)* | -0.01 (-0.23, 0.2) | 0.07 (-0.04, 0.17) | UC |  |
| 0.25 (0.16,0.34)* | 0.53 (0.30,0.75)* | 0.44 (0.31,0.57)* | 0.51 (0.43,0.59)* | Yoga |

*Means P < 0.05.

TableS8 SGRQ Schedule

| **MD (95%CrI)** | | | | | |
| --- | --- | --- | --- | --- | --- |
| Baduanjin |  |  |  |  |  |
| -7.72 (-21.89, 6.32) | Composite digital interventions |  |  |  |  |
| - 6.27 (-20.8, 7.95) | 1.43 (-15.13, 17.9) | Synchronous telerehabilitation |  |  |  |
| 0.69 (-10.35, 12.05) | 8.39 (-5.1, 22.37) | 6.99 (-6.89, 21.18) | Tai_Chi |  |  |
| - 8.44 (-16.76, -0.31)* | -0.73 (-12.25, 10.82) | -2.17 (-13.91, 9.63) | -9.16 (-16.89, -1.76)* | UC |  |
| -3.35 (-18.47, 11.81) | 4.36 (-12.65, 21.62) | 2.94 (-14.26, 20.3) | - 4.06 (-18.88, 10.73) | 5.08 (-7.5, 17.93) | Yoga |

*Means P < 0.05.

TableS9 CAT League Schedule

| **MD (95%CrI)** | | | | | |
| --- | --- | --- | --- | --- | --- |
| Baduanjin |  |  |  |  |  |
| 0.19 (-0.75, 1.13) | Synchronous telerehabilitation |  |  |  |  |
| 0.5 (-0.32, 1.31) | 0.31 (-0.66, 1.27) | Tai Chi |  |  |  |
| -1.54 (-2.46, -0.62)* | -1.73 (-2.79, -0.68)* | -2.04 (-2.99, -1.09)* | Composite digital interventions |  |  |
| -2.65 (-3.2, -2.1)* | -2.84 (-3.6, -2.08)* | -3.15 (-3.75, -2.55)* | -1.11 (-1.84, -0.37)* | UC |  |
| -2.50 (-4.01, -0.98)* | -2.68 (-4.29, -1.09)* | -2.99 (-4.52, -1.46)* | -0.95 (-2.53, 0.63) | 0.15 (-1.25, 1.56) | Yoga |

*Means P < 0.05.

TableS10 GRADE Assessment

| **Outcome** | **Comparison** | **Participants**  **(Studies)** | **Effect estimate**  **(MD, 95% CrI)** | **Risk of bias** | **Inconsistency** | **Indirectness** | **Imprecision** | **Publication bias** | **Certainty of evidence** |
| --- | --- | --- | --- | --- | --- | --- | --- | --- | --- |
| 6MWD | Yoga vs UC | 4720 (25 RCTs) | 62.71 m (17.58 to 105.19) | Serious | Serious | Not serious | Not serious | Undetected | ⨁⨁◯◯ Moderate |
| 6MWD | Baduanjin vs UC | 4720 (25 RCTs) | 55.34 m (23.82 to 86.65) | Serious | Serious | Not serious | Not serious | Undetected | ⨁⨁◯◯ Moderate |
| FEV₁ | Yoga vs UC | 4720 (31 RCTs) | 0.75 L (0.26 to 1.21) | Serious | Serious | Not serious | Not serious | Undetected | ⨁⨁◯◯ Moderate |
| FEV₁ | Baduanjin vs UC | 4720 (31 RCTs) | 0.37 L (0.13 to 0.63) | Serious | Serious | Not serious | Not serious | Undetected | ⨁⨁◯◯ Moderate |
| FVC | Baduanjin vs UC | 4720 (23 RCTs) | 0.26 L (0.22 to 0.30) | Not serious | Not serious | Not serious | Not serious | Undetected | ⨁⨁⨁⨁ High |
| SGRQ | Tai Chi vs UC | 4720 (15 RCTs) | −9.16 (−16.89 to −1.76) | Serious | Serious | Not serious | Not serious | Possible | ⨁⨁◯◯ Moderate |
| SGRQ | Baduanjin vs UC | 4720 (15 RCTs) | −8.44 (−16.76 to −0.31) | Serious | Serious | Not serious | Serious | Possible | ⨁◯◯◯ Low |
| CAT | Tai Chi vs UC | 4720 (20 RCTs) | −9.16 (−16.89 to −1.81) | Serious | Moderate | Not serious | Not serious | Possible | ⨁⨁◯◯ Moderate |
| CAT | Baduanjin vs UC | 4720 (20 RCTs) | −8.43 (−16.73 to −0.27) | Serious | Moderate | Not serious | Serious | Possible | ⨁◯◯◯ Low |
